# The Nontypeable Haemophilus influenzae Major Adhesin Hia Is a Dual-Function Lectin That Binds to Human-Specific Respiratory Tract Sialic Acid Glycan Receptors

**DOI:** 10.1128/mBio.02714-20

**Published:** 2020-11-03

**Authors:** John M. Atack, Christopher J. Day, Jessica Poole, Kenneth L. Brockman, Jamie R. L. Timms, Linda E. Winter, Thomas Haselhorst, Lauren O. Bakaletz, Stephen J. Barenkamp, Michael P. Jennings

**Affiliations:** a Institute for Glycomics, Griffith University, Gold Coast, Queensland, Australia; b Center for Microbial Pathogenesis, The Research Institute at Nationwide Children’s Hospital and The Ohio State University College of Medicine, Columbus, Ohio, USA; c Department of Pediatrics, Saint Louis University School of Medicine, and the Pediatric Research Institute, Cardinal Glennon Children’s Medical Center, Saint Louis, Missouri, USA; University of Pittsburgh School of Medicine

**Keywords:** COPD, NTHi, adhesin, autotransporter proteins, bacterial pathogen, glycan, host cell, middle ear infection

## Abstract

NTHi is a human-adapted pathogen that colonizes the human respiratory tract. Strains of NTHi express multiple adhesins; however, there is a unique, mutually exclusive relationship between the major adhesins Hia and HMW1 and HMW2 (HMW1/2). Approximately 25% of NTHi strains express Hia, a phase-variable autotransporter protein that has a critical role in colonization of the host nasopharynx. The remaining 75% of strains express HMW1/2. Previous work has shown that the HMW1 and HMW2 proteins mediate binding to 2-3- and 2-6-linked sialic acid glycans found in the human respiratory tract. Here, we show that the high-affinity binding domain of Hia, binding domain 1 (BD1), is responsible for binding to α2-6-sialyllactosamine (2-6 SLN) glycans. BD1 is highly specific for glycans that incorporate the form of sialic acid expressed by humans, *N*-acetylneuraminic acid (Neu5Ac). We further show that Hia has lower-affinity binding activity for 2-3-linked sialic acid and that this binding activity is mediated via a distinct domain. Thus, Hia with its dual binding activities functionally mimics the combined activities of the HMW1 and HMW2 adhesins. In addition, we show that Hia has a role in biofilm formation by strains of NTHi that express the adhesin. Knowledge of the binding affinity of this major NTHi adhesin and putative vaccine candidate will direct and inform development of future vaccines and therapeutic strategies for this important pathogen.

## Abstract (precis)

Host-adapted bacterial pathogens like NTHi have evolved specific mechanisms to colonize their restricted host niche. Relatively few of the adhesins expressed by NTHi have been characterized as regards their binding affinity at the molecular level. In this work, we show that the major NTHi adhesin Hia preferentially binds to Neu5Ac-α2-6-sialyllactosamine, the form of sialic acid expressed in humans. The receptors targeted by Hia in the human airway mirror those targeted by influenza A virus and indicates the broad importance of sialic acid glycans as receptors for microbes that colonize the human airway.

## INTRODUCTION

Nontypeable Haemophilus influenzae (NTHi) is a human-adapted pathogen responsible for multiple acute and chronic infections of the respiratory tract, including otitis media (OM) (1), community-acquired pneumonia (2), and chronic obstructive pulmonary disease (COPD) exacerbations (3). Each year, 31 million new cases of the most severe form of OM, chronic suppurative OM, are diagnosed (4), 60% of which suffer an associated hearing loss. Globally, there are over 700 million cases of acute OM every year (4); in the United States alone each year there are ∼25 million episodes of acute OM, >13 million antibiotic prescriptions, and public health costs estimated at $3 to $5 billion (5, 6). According to WHO estimates, approximately 65 million people have moderate to severe COPD. Over 3 million people died of COPD in 2005, which corresponded to 5% of all deaths globally (7). Invasive disease caused by NTHi has increased significantly in recent years, in part as a result of broad usage of vaccines against Haemophilus influenzae type b and Streptococcus pneumoniae (8). At present, there is no effective vaccine against NTHi.

NTHi is commonly carried asymptomatically within the human nasopharynx. Many bacterial pathogens express outer-surface proteins that target specific host molecules to allow them to adhere to and persist in specific niches within the host. Examples of bacterial adhesins recognizing particular host proteins include the type IV pilus of Neisseria gonorrhoeae, which recognizes host integrins (9); the type IV pilus of NTHi, which recognizes ICAM1 (10); the curli pili of Salmonella enterica, which bind host TLR2 receptors; and the FimH protein of uropathogenic Escherichia coli, which binds to mannosylated glycoproteins (11). Many bacteria also express virulence factors that belong to the autotransporter protein family. These proteins have a diverse array of functions, including adhesion to host surfaces (12). Autotransporter proteins are characterized by a large barrel-like C-terminal domain that inserts into the outer membrane, forming a pore through which the N-terminal effector portion passes to reach the extracellular environment (13, 14). NTHi expresses many autotransporter proteins (15) that fulfil a variety of roles in NTHi pathobiology. One of these autotransporters, Hia, is an adhesin that is expressed by approximately 25% of NTHi strains (16). The remaining ∼75% of NTHi strains express the HMW1 and 2 (HMW1/2) proteins (17), which have previously been demonstrated to be involved in adhesion of NTHi to human cells (18). It is unclear why strains encode genes for Hia or HMW but never both. The HMW1 protein binds to host cell glycans as cellular receptors, specifically α2-3-sialyllactosamine (2-3 SLN) (19). We recently demonstrated that HMW2, which is ∼65% identical to HMW1, binds the related glycan α2-6-sialyllactosamine (2-6 SLN), with high specificity for 2-6 SLN containing *N*-acetylneuraminic acid (Neu5Ac), the form of sialic acid expressed by humans (20). Intriguingly, 2-3 SLN is found mainly in the lower human respiratory tract, whereas 2-6 SLN is found throughout the entire respiratory tract but predominates in the upper airway (21). It has previously been demonstrated that Hia is required for adherence to Chang epithelial cells (22), and we have demonstrated that Hia is required for colonization of the host nasopharynx (23). However, the cellular receptor for Hia is currently unknown. We hypothesized that Hia may also recognize host-specific glycans found in the human respiratory tract. In the current study, we present an investigation to identify and characterize the Hia cellular receptor.

## RESULTS

### Hia is a lectin that recognizes Neu5Ac-α2-6-lactosamine with high affinity.

In order to determine whether Hia had glycan binding activity, we cloned and overexpressed Hia from NTHi strain R2866 in Escherichia coli BL21 cells. Heterologous overexpression of Hia in E. coli was used previously to investigate Hia binding activity (22). Hia overexpression was confirmed by Western blotting and whole-cell enzyme-limited immunosorbent assay (ELISA) (see Fig. S1 in the supplemental material). The glycan binding ability of E. coli strain BL21 cells expressing Hia (BL21-Hia) was compared to that of wild-type (wt) BL21 cells using glycan array analysis. The background binding of BL21 only was subtracted from BL21-Hia in order to deduce the glycans that bound in an Hia-dependent manner. A subset of the identified glycans were characterized for their binding affinity to BL21-Hia using surface plasmon resonance (SPR) (Table 1). These studies demonstrated that Hia bound to a number of sialylated glycans, with the greatest affinity for Neu5Ac-α2-6-lactosamine (2-6 SLN-Ac), with a disassociation constant (*K_D_*) of 185 nM. A comparison of the binding affinity of Hia to matched glycan pairs containing either a terminal *N-*acetylneuraminic acid (Neu5Ac; the only form expressed in humans) or *N-*glycolylneuraminic acid (Neu5Gc; expressed in most mammals), showed that Hia preferentially binds to structures containing a terminal Neu5Ac (Table 1), with an ∼7-fold preference for 2-6 SLN-Ac over Neu5Gc-α2-6-lactosamine (2-6 SLN-Gc) (185 nM versus 1.39 μM; Table 1). While some binding to 2-3 SLN-Ac (2.03 μM; Table 1) was observed, this occurred with approximately 11-fold lower affinity than that to 2-6 SLN-Ac (185 nM).

**TABLE 1:**
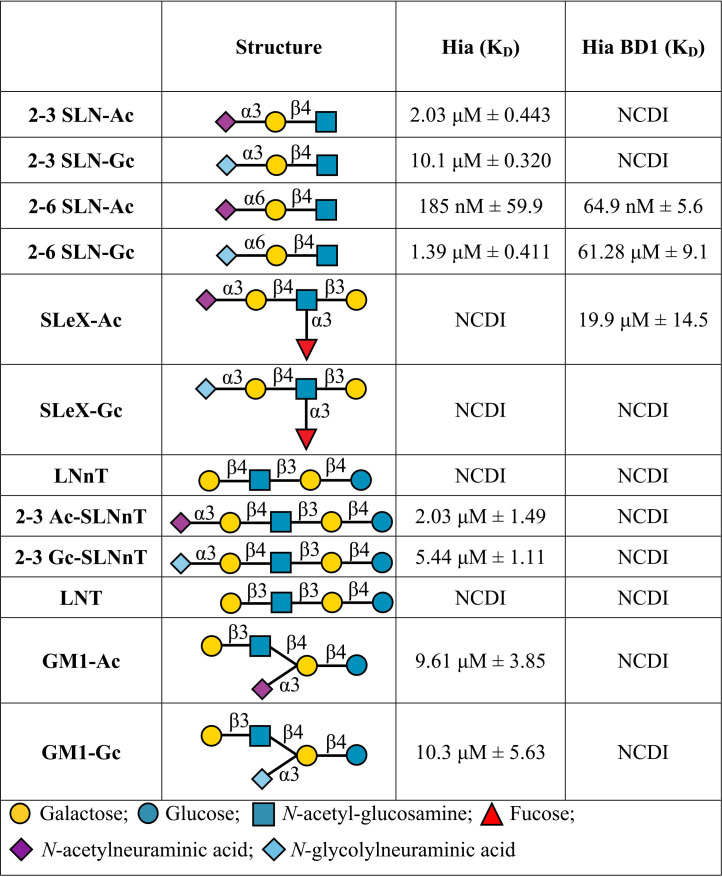
Surface plasmon resonance analysis of glycan binding affinity of BL21-Hia and purified recombinant Hia BD1^*a*^

a NCDI, no concentration-dependent interaction. Indicates no binding between Hia-expressing bacteria and the structure at a maximum concentration of 100 μM.

10.1128/mBio.02714-20.1 FIG S1 (A) Western blot showing the overexpression of wild-type Hia in Escherichia coli BL21. (B) Whole-cell enzyme-limited immunosorbent assay (ELISA) showing that Hia expressed in E. coli BL21 is located on the bacterial cell surface. Download FIG S1, PDF file, 1.6 MB. Copyright © 2020 Atack et al. 2020 Atack et al. This content is distributed under the terms of the Creative Commons Attribution 4.0 International license.

### Modeling shows key interactions between BD1 residues D618 and A620 and the Neu5Ac moiety of 2-6 SLN-Ac.

**FIG 1:**
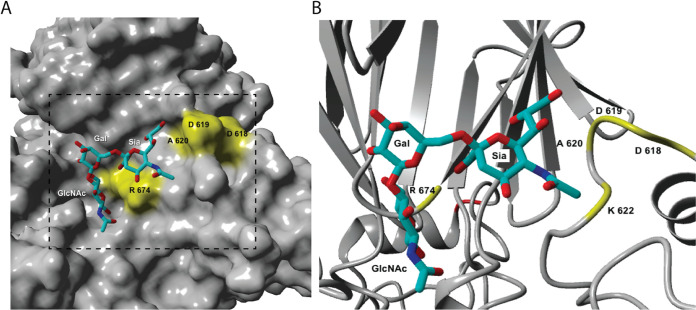
Molecular docking of Hia binding domain 1 (BD1) to 2-6 SLN. The previously published structure of Hia BD1 was used (PDB accession number 1S7M (22). Docking structure of 2-6 SLN-Ac into Hia (22). (A) Solid surface of 1S7M and (B) magnified region of 1S7M shown as a secondary structure and bound 2-6 SLN-Ac. Key amino acids are labeled.

The Hia protein has previously been shown to contain high- and low-affinity host cell binding domains (BD), termed BD1 and BD2, respectively (22, 24). BD1 and BD2 are proposed to bind a common but unknown cellular receptor (24). Hia BD1 consists of amino acids (aa) 541 to 714, inclusive (22), with residues in the BD1 shown to be essential for binding to Chang epithelial cells when Hia is expressed in E. coli (22). To determine the molecular basis of the interactions between Hia BD1 and 2-6 SLN-Ac, we carried out molecular docking studies using the previously published Hia BD1 structure (22). All docking structures of 2-6 SLN-Ac with Hia BD1 indicated interaction of the ligand at the interface of chain A and chain C of Hia BD1. Figure 1 shows a bound structure of 2-6 SLN-Ac that represents a sialic acid-specific binding mode with the negatively charged carboxylate group of the Neu5Ac residue engaging in strong electrostatic interaction with R674. The glycerol side chain of the sialic acid moiety of 2-6 SLN-Ac plays an important role, as it engages in hydrogen bonds with D618 and A620. Importantly, the high flexibility of the α(2-6) linkage of 2-6 SLN-Ac allows the coordination of the lactosamine disaccharide moiety. In addition, our docking studies indicate that residue R674 is involved in coordinating 2-6 SLN-Ac in all 25 potential docked conformations.

### Hia BD1 is the site of high-affinity interactions with the cellular receptor 2-6 SLN-Ac.

Using purified Hia BD1 (aa 514–714, inclusive) (22) we investigated BD1 binding specificity using SPR. Table 1 shows that Hia BD1 binds with high affinity and specificity to 2-6 SLN-Ac, with a *K_D_* of 64.9 nM ± 5.6. This value is in a similar range to the affinity we observe with full-length Hia (185 nM ± 59.9). Hia BD1 interacts with 2-6 SLN-Gc with ∼1,000-fold lower affinity (61.28 μM ± 9.1; see Table 1) than that with 2-6 SLN-Ac. In order to determine the specific region of BD1 responsible for the interaction with 2-6 SLN-Ac, we constructed a peptide library of BD1 aa 541 to 714 consisting of peptides of 15 amino acids in length and overlapping consecutive peptides by 10 amino acids each (for example, peptide 1 [p1] consisted of residues 541 to 555; peptide 2 of residues 546 to 560, etc.; Table 2). We used these peptides to block the interaction between BL21-Hia and 2-6 SLN-Ac using an SPR competition assay. Using this methodology, we show that a peptide comprised of 20 amino acid residues containing both D618 and A620 (p16 + 17; residues 616 to 635) blocks 100% of the interaction between BL21-Hia and 2-6 SLN-Ac (Table 2). Peptide 16 and peptide 17 individually result in blocking of 95% and 85% of interactions, respectively, between BL21-Hia and 2-6 SLN-Ac (Table 2). Peptides flanking the region of 16 and 17 (peptide 15 = aa 611 to 625; peptide 18 = aa 626 to 640; Table 2) only block ∼50% of interactions, with no other peptide 15-mer of BD1 blocking interactions between BL21 Hia and 2-6 SLN-Ac (data not shown). Residues D618 and A620 were previously shown to be key for binding to host cells, since when these residues were mutated (D618K and A620R), binding was lost (22). Our blocking studies provide strong evidence that additional residues, and likely secondary structures around these residues that can only form in the 20-mer comprising p16 + 17, mediate direct interaction between 2-6 SLN-Ac and Hia, leading to high-affinity binding.

**TABLE 2:** Surface plasmon resonance analysis of the blocking activity of peptides to interfere with the Hia:2-6 SLN-Ac interaction

Peptide	Sequence^*a*^	Blocking against 2-6 SLN-Ac
p15	DNLTKQN **D**D **A**YKGLT	54% ± 6.9%
p16	QN **D**D **A**YKGLTNLDEK	95% ± 4.8%
p17	YKGLTNLDEKGTDKQ	85% ± 8.6%
p18	NLDEKGTDKQTPVVA	48% ± 1.7%
p16/17 common	YKGLTNLDEK	95% ± 4.3%
p16 + 17	QN **D**D **A**YKGLTNLDEKGTDKQ	100% ± 2.5%

a Bold, underlined type indicates D618 and A620.

In order to confirm these findings, we generated recombinant Hia with the single mutations D618K and A620R and a double mutant of Hia lacking both of these residues (D618K/A620R double). SPR analysis was used to compare the binding of this panel of Hia mutants with that of wild-type Hia and BD1, using the same subset of glycans (see Table 3). These findings demonstrated that the A620R Hia mutant and the D618K/A620R Hia double mutant (all located in BD1) completely lose the ability to bind 2-6 SLN-Ac, while still maintaining binding to 2-3 SLN-Ac. Collectively, these data demonstrated that the binding site of 2-3 SLN-Ac is not BD1 and confirmed the role of BD1 in binding specificity to 2-6 SLN-Ac.

**TABLE 3:** Surface plasmon resonance analysis of glycan binding affinity of E. coli BL21 expressing wild-type Hia and Hia isogenic mutants

Hia type	2-3 SLN-Ac	2-6 SLN-Ac
Hia wild type	2.47 μM ± 0.8	110 nM ± 3.0
Hia D618K	842 nM ± 178	21 nM ± 1.0
Hia A620R	3.04 μM ± 0.7	NB^*a*^
Hia D618K/A620 double	3.42 μM ± 1.2	NB

a NB, no binding using a OneStep injection at 10 μM; indicates a *K_D_* above 10 μM.

### Hia is involved in interactions between NTHi and epithelial cells.

**FIG 2:**
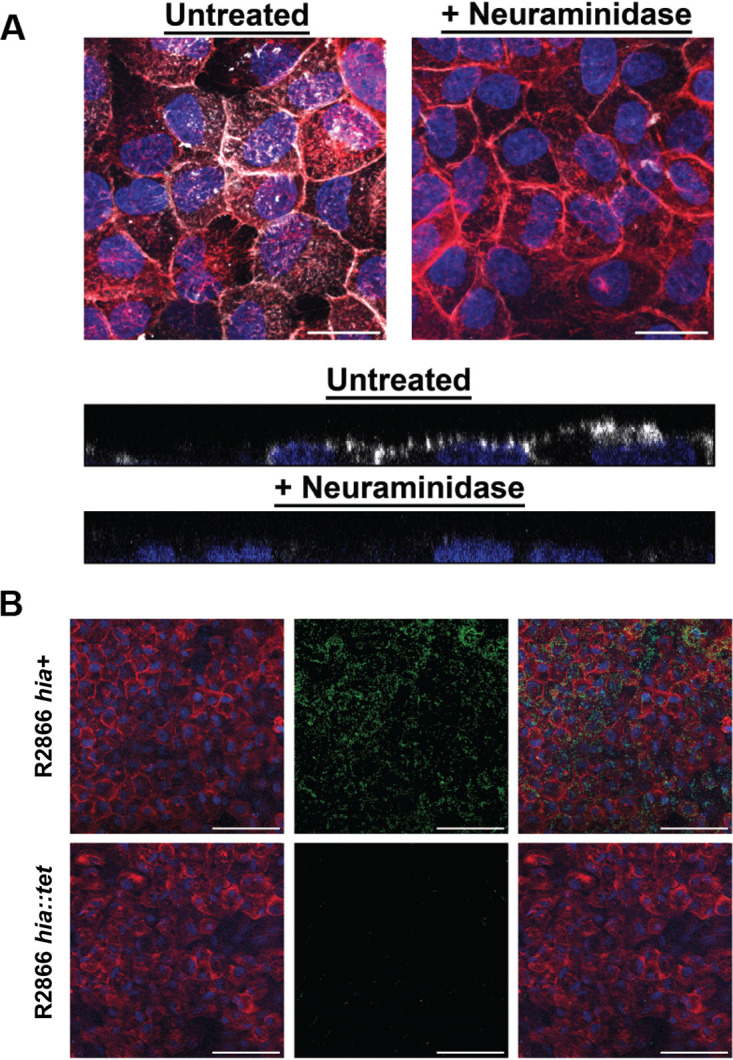
2-6 SLN presence on Chang cells and adherence of NTHi strain R2866 expressing Hia to Chang cells. (A) 2-6 Sialyl-*N*-acetyllactosamine expressed on the surface of Chang cells. (Upper) Top-down view of 2-6 SLN distribution on Chang cells or cells pretreated with neuraminidase. White, SNAi; red, phalloidin; blue, nuclear DNA. Bar, 25 μm. (Lower) Representative side view of an optical section through SNAi-labeled Chang cells. White, SNAi; blue, nuclear DNA. (B) Chang cell R2866 adherence. Adherence of wild-type R2866 expressing Hia (*hia*^+^) and the R2866 *hia*::*tet* mutant to Chang cells. (Left) Chang cell monolayer with phalloidin shown in red and nuclear DNA shown in blue. (Middle) Distribution of strain R2866 mutants that constitutively express green fluorescent protein (GFP), shown in green. Bacteria that express Hia (*hia*+) bound markedly better to Chang cells than those that do not express Hia (*hia*::*tet*). (Right) Merged images that show distribution of strain R2866 mutants across the surface of the Chang cells. Bar, 100 μm.

In order to demonstrate a biological role for Hia in attachment of NTHi to host epithelium, we performed adherence assays using Chang epithelial cells. Prior to carrying out these adherence assays, we confirmed 2-6 SLN was localized on the surface of these cells using Dylight 649-conjugated *Sambucus nigra* lectin (SNA), a lectin specific for 2-6 SLN (Fig. 2A). Following treatment with sialidase to remove sialylated glycans, 2-6 SLN was no longer detected on the cell surface by SNA (Fig. 2A). Using NTHi strain R2866 that expressed Hia (wild type R2866; R2866 *hia*^+^ strain), and an isogenic mutant lacking Hia (R2866 *hia*::*tet* strain), we showed that the ability of NTHi to adhere to Chang cells decreased when NTHi lacked Hia. Wild-type R2866 is unable to bind Chang cells treated with sialidase, which removes sialylated glycan structures (Fig. 2A).

### Residues D618 and A620 are critical to the interaction of Hia with Chang cells.

**FIG 3:**
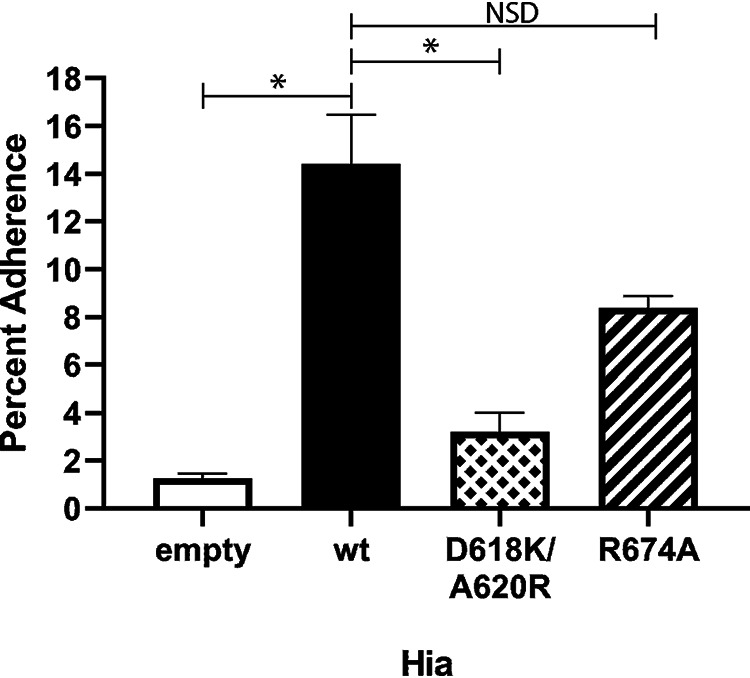
Percent adherence to Chang cells of E. coli BL21 strains expressing wild-type Hia or isogenic mutant variants. Percent adherence of each strain is calculated as adherent CFU following 2 h of incubation divided by total input CFU. All raw data are presented in Data Set S1 in the supplemental material. *, *P* < 0.005. NSD, no significant difference. *P* values were calculated using one-way analysis of variance (ANOVA).

In order to determine the contribution of the key 2-6 SLN-Ac interacting residues (D618 and A620) and residue R674, indicated as important from our modeling studies, we carried out adherence assays using a Chang epithelial cell model (23) to determine relative adherence of E. coli BL21 strains expressing wild-type (wt) Hia and our panel of Hia point mutants. Adherence of E. coli BL21 cells to Chang cells was significantly greater when cells expressed wt Hia (14.44% adherence; Fig. 3) compared to control cells that did not express Hia (empty BL21; 1.26% adherence; *P* = 0.0007). BL21 cells that expressed the Hia D618K/A620R double mutant exhibited an approximately 4.5-fold decrease in relative adherence (3.21% adherence; *P* = 0.002) compared to BL21 cells that expressed wt Hia. BL21 that expressed Hia R674A showed an approximate 2-fold decrease in relative adherence compared to cells that expressed wt Hia (8.4% adherence), but this was not statistically significant compared to cells expressing wt Hia (*P* = 0.06). These data indicate that the interaction between Hia and 2-6 SLN-Ac is critical to bacterial interactions with epithelial cells and demonstrate the key contribution of residues D618 and A620 of Hia BD1 in mediating this interaction.

10.1128/mBio.02714-20.6 DATA SET S1 CFU counts from adherence assays with Chang cells used to plot. Download Data Set S1, XLSX file, 0.01 MB. Copyright © 2020 Atack et al. 2020 Atack et al. This content is distributed under the terms of the Creative Commons Attribution 4.0 International license.

### Expression of Hia in NTHi results in larger, denser biofilms.

**FIG 4:**
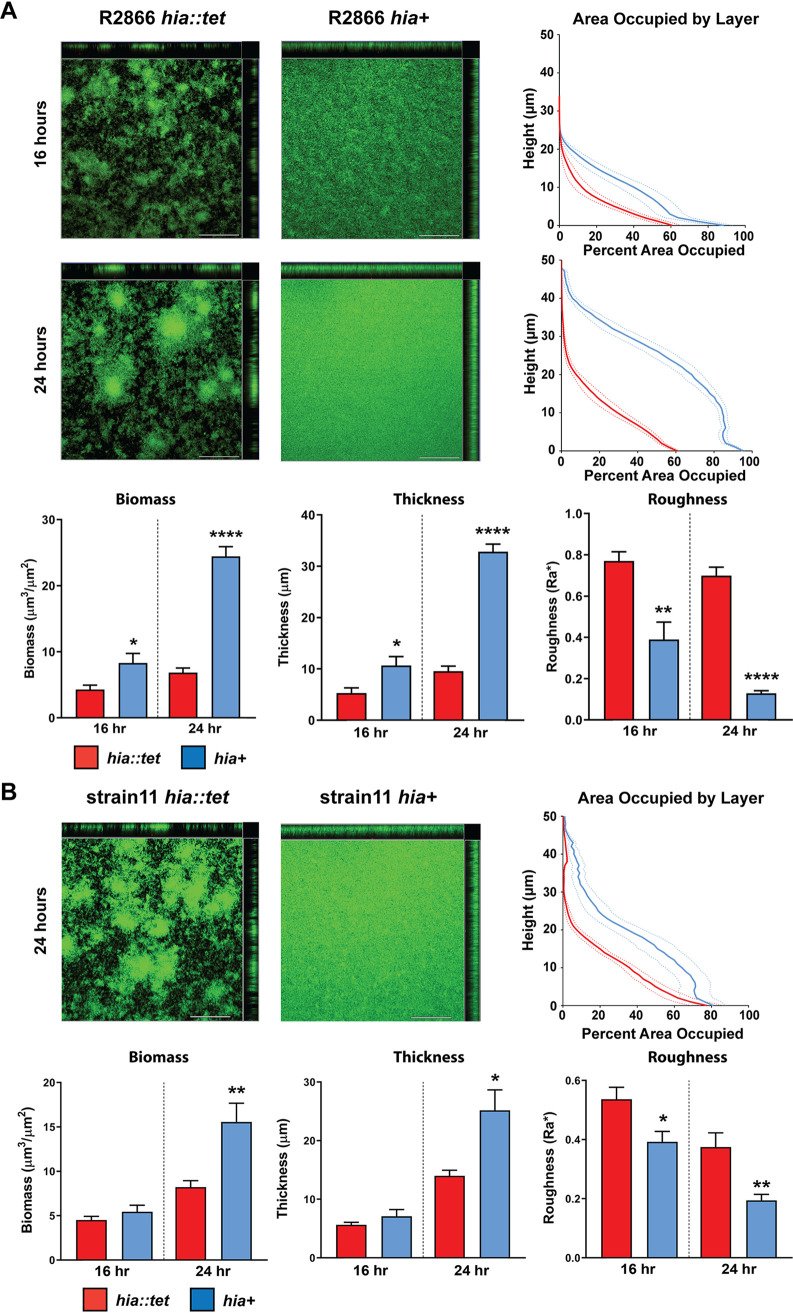
Biofilm formation by NTHi strains R2866 and strain 11. Representative orthogonal image renderings of biofilm formation by NTHi strain R2866 and strain 11 *hia*::*tet* and *hia*^+^ biofilms. Bars, 100 μm. Biomass, average thickness, and roughness of *hia*::*tet* and *hia*^+^ biofilms grown for 16 or 24 h were analyzed by COMSTAT2, and values are shown as mean ± standard error of the mean. *, P < 0.05, **, P < 0.01, ****, P < 0.0001; one-way ANOVA with Dunnett’s *post hoc* test. Average percent area occupied by bacteria at each individual 1-μm optical section (“layer”) were determined with COMSTAT2. Dashed lines indicate standard error of the mean.

The role of Hia in biofilm formation by two NTHi strains (R2866 and strain 11, both encoding the *hia* gene) was tested using our well-defined static biofilm model for NTHi (25). Biofilms were formed for up to 24 h at 37°C. After 24 h, both strain R2866 and strain 11 formed much larger biofilms when Hia was expressed (*hia*^+^) compared to when it was absent, as assessed by confocal microscopy (Fig. 4). After 24 h, NTHi that expressed Hia (*hia*^+^) formed biofilms with significantly more biomass (*P* < 0.0001 for strain R2866 [Fig. 4A] *P* < 0.01for strain 11 [Fig. 4B]) and were significantly thicker (*P* < 0.0001 for strain R2866 Fig. 4A; *P* = <0.05 strain 11; Fig. 4B) compared to those formed by strains that did not express Hia (*hia*::*tet*). Descriptively, biofilms formed by NTHi that expressed Hia were significantly denser, and had a lawn-like architecture, compared to those formed by the respective isogenic mutant strain that did not express Hia. Biofilms of the two *hia*::*tet* isogenic mutant strains had a greater difference in overall surface height and topography, with dense tower-like regions of bacteria surrounded by open water channels (indicated by black in the representative top-down images) (Fig. 4). The differences in bacterial distribution within the biofilm are apparent from the area occupied by layer (AOL) graphs (Fig. 4). The area occupied by layer is a calculation of the amount (or percentage) of bacterial biomass that is present within each 1-μm optical section of the biofilm taken from the base of the biofilm to the top. These data are plotted such that the layer closest to the glass surface is at the bottom of the *y* axis, and the top of the biofilm (farthest from the surface) is at the top of the *y* axis. The relative shift of the blue lines to the right and upward, compared to the red lines (Fig. 4), indicated that biofilms formed by NTHi that express Hia are substantially denser and have greater biomass than those formed by NTHi that do not express Hia. Biofilms formed by strain R2866 for only 16 h were smaller than those at 24 h but with similar differences in biofilm size and architecture with respect to Hia expression. R2866 expressing Hia (*hia*^+^) formed a biofilm with greater biomass (*P* < 0.05; Fig. 4A) and average thickness (*P* < 0.05; Fig. 4A) compared to the isogenic knockout strain (*hia*::*tet*). Biofilms formed by the *hia*^+^ strain had a lawn-like appearance that more confluently covered the substratum, while the *hia*::*tet* mutant had much more heterogeneous distribution, with visible tower-like structures (Fig. 4A). Interestingly, strain 11 did not display the same phenotype after 16 h, with both the *hia*^+^ and *hia*::*tet* strains displaying biofilms of similar size at this time point (no significant difference between biomass or average thickness; Fig. 4B). To identify Hia-mediated differences in the very initial stages of biofilm formation and attachment, biofilms formed by strain R2866 for only 6 h were analyzed. At this early time point, no significant differences in biomass, thickness, or roughness were observed between the *hia*^+^ and *hia*::*tet* strains; however, the biofilms were substantially smaller than those formed after 16 or 24 h of growth and did show a similar trend (see Fig. S2 in the supplemental material). These data suggest that the observed differences in biofilm formation are not solely due to initial attachment to the glass surface but also to Hia affecting bacterial cell-cell interactions during biofilm development. Strain-specific differences at the 16-h time point are likely due to determinants other than Hia, which highlights that while Hia plays a key role in biofilm formation by NTHi, other factors are involved as well. These results indicated that the Hia adhesin was a critical determinant of biofilm structure and organization in these strains, possibly due to increased interbacterial associations.

10.1128/mBio.02714-20.2 FIG S2 Six-hour biofilm formation by NTHi strain R2866. Representative orthogonal image renderings of biofilms formed for 6 h by NTHi R2866 *hia*::*tet* and *hia*^+^ strains. Bars, 100 μm. Biomass and average thickness and roughness of *hia*::*tet* and *hia*^+^ biofilms were analyzed by COMSTAT2, and values are shown as mean ± standard error of the mean. Average percent area occupied by bacteria at each individual 1-μm optical section (“layer”) were determined. Dashed lines indicate standard error of the mean. No significant difference between *hia*::*tet* and *hia*^+^ biomass (*P = *0.0156), roughness (*P = *0.019806), or thickness (*P = *0.213082) was detected using one-way analysis of variance (ANOVA). Download FIG S2, JPG file, 2.6 MB. Copyright © 2020 Atack et al. 2020 Atack et al. This content is distributed under the terms of the Creative Commons Attribution 4.0 International license.

## DISCUSSION

In this work, we demonstrated that the NTHi adhesin Hia is a lectin, with high specificity for host-specific glycans. Hia mediates high-affinity binding to 2-6 SLN-Ac. Molecular modeling studies using the crystal structure of Hia BD1 (22) in complex with 2-6 SLN-Ac showed that Hia residues D618 and A620, and to some extent R674, were critical to this interaction. We experimentally confirmed our modeling using a diverse and comprehensive array of complementary *in vitro* studies. Using a combination of E. coli expressing Hia, purified Hia BD1, and a peptide library derived from BD1, we determined that residues D618 and A620 of Hia are required for the high-affinity interaction between Hia and 2-6 SLN-Ac. Interestingly, our SPR data also confirmed that Hia recognizes 2-3 SLN-Ac, but this interaction was approximately 10-fold lower than that for 2-6 SLN-Ac. However, using the Hia BD1 protein, we confirmed that the interaction of Hia with 2-3 SLN-Ac is not mediated by BD1, which is consistent with previous findings that proposed that Hia contains two binding domains (22). Therefore, contrary to previous work which stated that BD1 and BD2 bind the same ligand (24), we have shown that the two binding domains of Hia interact with distinct ligands, BD1 with 2-6 SLN-Ac and BD2 with 2-3 SLN-Ac. Moreover, in both cases, the preference is for the form of sialic acid expressed by humans (Neu5Ac).

The binding preference of Hia BD1 for Neu5Ac offers an insight into the evolution of NTHi as a human-specific pathogen: although Neu5Gc and Neu5Ac (the precursor to Neu5Gc) can be expressed by most mammals, humans only make Neu5Ac-linked glycans, due to a mutation in the CMAH gene responsible for the conversion of Neu5Ac to Neu5Gc (26). Hia preferentially binds Neu5Ac linked glycans over Neu5Gc linked glycans. This finding strongly suggests that Hia has evolved to preferentially bind glycans most likely to be present in its human host. Although NTHi can uptake and utilize both Neu5Ac and Neu5Gc equally as a carbon source, the Neu5Ac form of sialic acid is preferred for macromolecular biosynthesis of bacterial cell surface glycans by NTHi and has been proposed as an example of a human adaptation to achieve molecular mimicry, which in turn promotes immunoevasion (27). In a similar way, the high-affinity binding of the Hia adhesin for the only sialic acid that is expressed by humans, i.e., Neu5Ac, appears to be a similar adaptation. The chinchilla model of otitis media (OM) is recognized as the best animal model system to study OM caused by NTHi, as there are indeed many important parallels between experimental disease in the chinchilla and that which occurs in a human child (28). However, no animal model can reproduce every aspect of host-pathogen interactions for this exquisitely human-adapted pathogen. Chinchillas, like most mammals (29) express Neu5Gc as the predominant sialic acid, and therefore use of the chinchilla model system to specifically probe the role of Hia in NTHi pathogenesis is not appropriate, as they do not express the cognate Hia BD1 Neu5Ac receptor.

**FIG 5:**
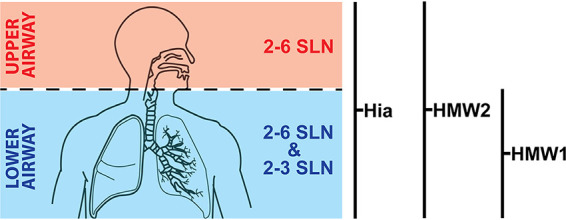
Illustration showing distribution of 2-6 SLN and 2-3 SLN in the human airway. Hia and HMW2 both bind 2-6 SLN with high affinity, with both showing a very high preference for the human-specific sialic acid Neu5Ac (2-6 SLN-Ac) over Neu5Gc (2-6 SLN-Gc). This means that NTHi strains expressing either Hia or HMW2 are able to colonize the entire respiratory tract (upper in red, lower in blue). HMW1 preferentially binds 2-3 SLN, and with no affinity for Neu5Ac over Neu5Gc, which means that NTHi strains only expressing HMW1 may have a preference for the lower respiratory tract (blue). Schematic diagram taken from GetDrawings.com (http://getdrawings.com/respiratory-system-with-label-drawing#respiratory-system-with-label-drawing-13.jpg) under a CC BY-NC 4.0 license.

NTHi strains that do not possess the gene encoding *hia* instead encode genes for and express the adhesins HMW1 and HMW2 (18). Previous work has demonstrated that NTHi strains either encode genes for Hia or HMW1/2, but never both, with approximately 75% of strains expressing HMW1/HMW2 and the remaining 25% expressing Hia. We recently demonstrated that HMW2 preferentially binds 2-6 SLN-Ac (20), whereas HMW1 has a preference for 2-3 SLN structures (19). However, HMW1 showed no preference for either Neu5Ac- or Neu5Gc-containing structures and had a much lower affinity than HMW2 for 2-6 SLN-Ac (20). Therefore, two distinct NTHi adhesins, Hia and HMW1/2, that show a discrete lineage distribution in the NTHi population, have evolved to bind the same subset of glycans. Hia binds 2-6 SLN-Ac preferentially over 2-3 SLN-Ac; HMW2 specifically binds 2-6 SLN-Ac; HMW1 binds a broader range of 2-3- and 2-6-linked glycan structures than HMW2, but with lower overall affinity (Fig. 5). Production of both 2-6 SLN-Ac- and 2-3 SLN-Ac-linked glycan structures is found throughout the human airway, but these receptors are not evenly distributed throughout the upper and lower airway; 2-6 SLN-Ac is found in both the upper and lower respiratory tract, while 2-3 SLN-Ac-linked glycans are found predominantly in the lower respiratory tract (30–32). Thus, NTHi strains that express Hia or HMW1/2 are able to adhere to the entire human respiratory tract. It would be interesting to study the distribution of NTHi strains expressing only HMW1 or HMW2; based on their differing binding affinities, it may be that strains expressing HMW2 only are more prevalent in upper respiratory tract infections, whereas strains expressing HMW1 only are more disposed to infecting the lower respiratory tract. It is also intriguing to note that the binding specificity of Hia (and HMW1/2) mirrors perfectly that of human influenza A viruses (29, 33, 34), indicating that specificity for human-specific glycans has evolved in both viral and bacterial human-adapted airway pathogens. A common strategy to block these interactions may therefore serve as a general therapy for both these types of infection. It is well known that infection with the influenza virus predisposes individuals to colonization and infection by S. pneumoniae. Although there are likely multiple aspects behind the increased severity of pneumococcal disease following influenza virus infection (35), it is thought that desialylation of the host epithelia by the viral neuraminidase allows for more efficient colonization by the pneumococcus (36). This desialylation in turn increases the susceptibility of these patients to pneumococcal pneumonia following influenza virus infection. The lethality of the 1918 “Spanish flu” outbreak was mainly due to secondary infections by bacterial pathogens, including both S. pneumoniae and H. influenzae, with up to 95% of the deaths from this pandemic attributable to secondary bacterial infections (37, 38). Thus, the sharing of common receptors indicates the possibility of direct interaction between influenza virus and NTHi occurring during coinfection, and it may provide a fruitful area of investigation in the study of the dynamics of this polymicrobial interaction.

As well as Hia playing a key role in host colonization through recognition of human-specific glycan structures, we have demonstrated that Hia also plays a key role in biofilm formation by NTHi. Biofilm formation by NTHi has been shown to increase the resistance of bacteria to antibiotics (39) and killing by neutrophils (40) compared to that of planktonic counterparts. Increased resistance of bacteria within biofilms to antibiotics has been demonstrated for a number of major human pathogens, such as Pseudomonas aeruginosa (41) and Staphylococcus aureus (42). Biofilm formation also plays a key role in NTHi disease pathologies, such as middle ear infections (43) and exacerbations of cystic fibrosis (44). NTHi strains also directly incorporate sialic acid into their lipooligosaccharide (LOS) (45), with the presence of these sialyl moieties on LOS promoting biofilm formation by NTHi (46, 47). Using two diverse strains of NTHi, we showed that Hia is a critical determinant of biofilm development and structure and that the potential to block Hia function through knowledge of its specific binding affinities could play a key role in targeting biofilm formation during disease caused by NTHi. It could be that Hia directly binds to the sialic acid found on LOS to mediate bacteria-bacteria interactions and enhance the formation and stability of NTHi biofilms, in addition to mediating adherence directly to host cells, but this would require experimental validation. In addition, differences in biofilm structure, such as those observed between *hia^+^* and *hia*::*tet* strains, could alter diffusion of nutrients and antimicrobials into the biofilms, as well as impede access of phagocytic immune cells to bacteria within the biofilm. To summarize, we have provided an in-depth characterization of the binding affinity of the NTHi adhesin Hia by determining the major human cellular receptors it has evolved to bind and by demonstrating the molecular basis of these interactions. We also demonstrate that Hia has a role in biofilm formation by NTHi and therefore likely contributes to antibiotic resistance and chronicity by this mechanism. Knowledge of the factors required by NTHi to colonize and cause disease will be key to developing both vaccines and treatments against this organism. Our demonstration that the major NTHi adhesins HMW1 and HMW2 bind the same host glycans as Hia (20) and that these adhesins are expressed by nearly 100% of NTHi strains is a key step toward the development of a rationally designed vaccine against NTHi, and to the production of novel treatments against this pathogen.

## MATERIALS AND METHODS

### Bacterial strains and growth conditions.

NTHi strains expressing Hia have been described previously (R2866 [48] and strain 11 [49]). NTHi strains were routinely grown in brain-heart infusion (BHI) broth supplemented with 1% hemin and 20 μg NAD^+^/ml (sBHI) and grown aerobically at 37°C with 150 rpm shaking. For solid medium, 1.5% agar was added to sBHI broth. sBHI media were supplemented with tetracycline (5 μg/ml) as required. Plates were grown at 37°C in atmosphere containing 5% CO_2_. Escherichia coli cultures were grown using Luria-Bertani (LB) media at 37°C and supplemented with tetracycline (5 μg/ml) as required.

### Generation of an *hia* knockout mutant in NTHi strains R2866 and 11.

A region of the NTHi R2866 chromosome containing the *hia* promoter and the ATG start and 5′ region of the gene were generated by PCR using primer pair hia-UP-F/hia-UP-R and cloned into pGEM Teasy according to manufacturer’s instructions (Promega) to generate plasmid vector Teasy::hiaUP. Inverse PCR was used to linearize this vector at the *hia* start codon using primers hia-INV-F/hia-INV-R. A tetracycline resistance cassette, encoding TetM, was generated from plasmid vector pGEM-TetM(B) using M13F and M13R primers. This was cloned into the linearized Teasy::hiaUP vector so the gene was in the same orientation as the *hia* gene, and orientation confirmed using PCR and sequencing. This vector was designated Teasy::hiaUP::TetM. Following linearization with NgoMIV (New England Biolabs), DNA was transformed into NTHi strains R2866 and strain 11 using the MIV method (50). Transformants were selected on BHI medium containing 5 μg tetracycline/ml, and positive colonies were confirmed by sequencing and Western blotting using an anti-Hia monoclonal antibody 1F4 (51). Strains were designated R2866 or strain 11 *hia*::*tet* strain.

### Cloning and overexpression of full-length Hia in E. coli.

Primers HiaFULL-F and HiaFULL-R (see Table S1 in the supplemental material) were used to amplify full-length wild-type *hia* (R2866_0725) including the signal sequence (residues 1 to 49) from genomic DNA prepared from NTHi strain R2866. PCR was carried out using KOD hot-start polymerase (EMD Millipore) according to manufacturer’s instructions. Following digestion with BspHI and XhoI (NEB) and clean up, DNA was cloned into pET15b digested with NcoI and XhoI. The resulting plasmid was designated pET15b::Hia. Following confirmation of correct clones by sequencing, overexpression was carried out in E. coli BL21 following by inducing cells with 0.5 mM isopropyl-β-d-thiogalactopyranoside (IPTG) overnight at 37°C with 200 rpm shaking. Overexpression was confirmed by Western blotting as previously described (23) using anti-Hia monoclonal antibody 1F4 (51). Whole-cell ELISA using standard methods (52) with modifications as previously described (23) and starting with 1:10,000 dilution of primary antibody anti-Hia monoclonal antibody 1F4 confirmed the location of Hia at the cell surface.

10.1128/mBio.02714-20.3 TABLE S1 Primers used in this study. Download Table S1, DOCX file, 0.01 MB. Copyright © 2020 Atack et al. 2020 Atack et al. This content is distributed under the terms of the Creative Commons Attribution 4.0 International license.

### Generation of Hia point mutants for overexpression.

Inverse PCR was carried out using primer pairs designed to introduce point mutations as previously described and used here to abrogate binding of E. coli expressing Hia to Chang cells (22). D618K, A620A, and a 618/620 double mutant were generated using specific forward primers Hia-D618K-F, Hia-A620R-F, or Hia-618/620-double-F and common reverse primer Hia-618/620-R. A R674A mutant was generated using primer pair Hia-R674A-F and Hia-R674A-R. All inverse PCRs were carried out using KOD hot-start polymerase (EMD Millipore) according to manufacturer’s instructions, and a plasmid miniprep (Qiagen) of pET15b::Hia as the template. All primer sequences are listed in Table S1. Clones were sequenced using primers either side of the point mutation Hia-screen-F and Hia-screen-R using BigDye 3.1 according to manufacturer’s instructions (Thermo Fisher), and sequenced at Australian Genome Analysis Facility (AGRF, Brisbane, Australia). Overexpression was carried out as described above for the Hia wild type, and cell surface localization was confirmed using whole-cell ELISA as above.

### Overexpression and purification of Hia BD1.

Primers to clone Hia binding domain 1 (BD1; amino acid residues 540 to 714) were designed based on those from (22). HiaBD1-F and HiaBD1-R were used to amplify BD1 from NTHi strain R2866 genomic DNA using KOD hot-start polymerase (EMD Millipore) according to manufacturer’s instructions. Following digestion with NdeI and BamHI (NEB) and clean up, DNA was cloned into pET15b digested with the same enzymes. This strategy would clone the gene in frame with an N-terminal 6×His tag for purification. The resulting plasmid was designated pET15b::HiaBD1. Overexpression was carried out in E. coli BL21 following by inducing cells with 0.5 mM IPTG overnight at 37°C with 200 rpm shaking. Cells were pelleted, resuspended in 1× binding buffer (50 mM NaPO_4_ and 300 mM NaCl [pH 7.4]), lysed using 0.1-mm glass beads and a TissueLyser (Qiagen) for 30 min at 50 osc^−1^· min^−1^. Purification was carried out using Talon gravity flow resin in 1× binding buffer. Protein was eluted from the resin using stepwise concentrations of imidazole in 1× binding buffer (10 to 500 mM imidazole), fractions analyzed by SDS-PAGE, and fractions containing pure BD1 pooled and concentrated using centrifugal concentrators (10 kDa cutoff; Millipore). Pure concentrated BD1 was buffer exchanged into 1× phosphate-buffered saline (PBS) using the same centrifugal concentrators. Protein was analyzed by SDS-PAGE, and quantified using an extinction coefficient of 8480 M^−1^ · cm^−1^ and molecular weight (MW) of 20,471.43 Da (based on the sequence of Hia BD1 + 6×His tag).

### Glycan array.

Glycan array slides were printed using OPEpoxy (CapitalBio) activated substrates with the glycan library as previously described (53) using an ArrayIt Spotbot Extreme 3 contact printer with solid metal pins. The glycan array binding experiments were performed and analyzed as previously described (54). Briefly, 1 ml of optical density at 600 nm (OD_600_) 0.2 E. coli BL21 cells with heterologous expression of the Hia wild type or Hia point mutants in PBS were incubated with 15 μl of 50 μM Bodipy methyl ester for 15 min, centrifuged at 900 × *g* for 3 min and the pellet washed 3 times with PBS to removed excess dye. The cell pellet was resuspended in 1 ml of array PBS (1× PBS containing 1 mM CaCl_2_ and 1 mM MgCl_2_), and 300 μl was applied to the slide in a 65-μl gene frame without a coverslip. Slides were washed three times for 2 min in array PBS, dried by centrifugation, and scanned and analyzed using the Scan Array Express software package (Perkin Elmer) and Microsoft Excel for statistical analysis. Binding of Hia was defined as both above the background of the slide (cutoff, 550 fluorescence units) and 2-fold and significantly (*P < *0.05) above the background of empty vector BL21 binding to the array by Student’s unpaired *t* test of fluorescence of BL21 empty-vector controls versus Hia-expressing strains of BL21. All glycan array binding data are presented in Table S2, and the MIRAGE-compliant information is listed in Table S3 in the supplemental material.

10.1128/mBio.02714-20.4 TABLE S2 Glycan array results using BL21 expressing Hia wild type (wt) versus empty BL21. Red color indicates >2-fold binding above that of the BL21-only background. Download Table S2, PDF file, 0.2 MB. Copyright © 2020 Atack et al. 2020 Atack et al. This content is distributed under the terms of the Creative Commons Attribution 4.0 International license.

10.1128/mBio.02714-20.5 TABLE S3 Supplementary glycan microarray document based on MIRAGE guidelines (https://doi.org/10.1093/glycob/cww118). Download Table S3, DOCX file, 0.02 MB. Copyright © 2020 Atack et al. 2020 Atack et al. This content is distributed under the terms of the Creative Commons Attribution 4.0 International license.

### Surface plasmon resonance.

Surface plasmon resonance (SPR) experiments of the full-length wild-type Hia expressed on the surface of E. coli BL21 cells was performed using a GE Biacore T100 system and a series S C1 sensor chip using a modification of previously described methods (55, 56). E. coli (BL21 strains expressing full-length wild-type Hia, point mutants, or BL21 only) cells at 1 × 10^6^ bacteria/ml were immobilized to the chip surface following the C1 NHS/EDC method template with a contact time of 900 s at a flow rate of 5 μl/minute in 10 mM sodium acetate (pH 5.5). Interaction of glycans with the bacteria was performed using 5-fold serial dilutions, with maximum concentration of 20 μM on first analysis and 5 μM when affinities were better defined, using single-cycle kinetics in 1× PBS (pH 7.4) at 20 μl/minute with a 60-s contact time and a final dissociation time of 10 min. A blank ethanolamine immobilization was used as a control flow cell, and 1× PBS (pH 7.4) was used as the zero-concentration control. Regeneration of the bacterial surface was performed by flushing 10 mM Tris 1 mM EDTA over the surface for 5 min at 30 μl/minute. Affinities (*K_D_*) were determined using the Biacore T100 evaluation software analysis of double baseline subtracted data. All interactions were measured in triplicate and displayed plus/minus 1 standard deviation of the measured mean.

Purified Hia BD1 protein was immobilized onto a CM5 sensor chip amine capture on a Biacore T100 with a contact time of 600 s at a flow rate of 5 μl/minute in 10 mM sodium acetate (pH 4.5). Glycans were run at the optimized concentrations outlined above with the analysis performed as outlined above.

Peptide binding region identification was performed using a modified version of a previously described method (57), namely, competition assays using immobilized Hia-expressing cells and flowed peptides and glycan. E. coli BL21 expressing full-length wild-type Hia were immobilized onto an H1 sensor chip using a ForteBio Pioneer using a contact time of 720 s at a flow rate of 10 μl/minute in 1× PBS at 1 × 10^8^ bacteria/ml. Assays were set up using the NextStep injection feature as previously described (58, 59) with combinations of 2-6 SLN, Hia-overlapping peptides, and PBS as the negative control used to determine the Hia region that interacted with 2-6 SLN. Analysis was performed using the QDat analysis software package.

### Distribution of 2-6 SLN on Chang cells.

Chang cells (1 × 10^4^ cells) in 100 μl total volume were seeded into Transwell inserts with a 6.5-mm diameter and 0.4-μm pore size (Corning Incorporated, Corning, New York). Cell culture medium (Dulbecco’s modified Eagle’s medium [DMEM], 10% heat-inactivated calf serum, 2 mM l-glutamine) was replaced daily until cells reached confluence at 2 to 3 days. The apical surface of the cells was rinsed twice with sterile Dulbecco’s phosphate-buffered saline (DPBS) and then incubated with 0.1 units of neuraminidase (Sigma) in 100 μl DPBS or with DPBS alone for 2 h at 37°C. The cells were then rinsed twice with DPBS, and 100 μl of 10 μg/ml Dylight 649 conjugated Sambucus nigra (EY Laboratories, San Mateo, CA) was added to the apical surface and incubated for 15 min. The cells were rinsed twice with DPBS, incubated with 3 units of Alexa Fluor 594 phalloidin (Thermo Fisher Scientific, Waltham, MA) for 30 min and rinsed twice. The membrane of the Transwell was excised and then mounted with ProLong glass antifade mountant with NucBlue stain (Thermo Fisher Scientific, Waltham, MA). Images were captured on a LSM 700 laser scanning microscope and rendered with Zen software (Zeiss).

### Biofilm formation.

Biofilms were formed by NTHi cultured within chambers of eight-well-chambered cover glass slides (Thermo Scientific, Waltham, MA) as described previously (60). Briefly, mid-log-phase cultures of NTHi strains were diluted with sBHI. NTHi strains were inoculated at 4 × 10^4^ CFU in 200 μl total volume per well, and slides were incubated at 37°C with 5% atmospheric CO_2_. Biofilms were grown for up to 24 h, with the growth medium replaced after 16 h when applicable. To visualize, biofilms were stained with live/dead BacLight stain (Life Technologies) and fixed overnight in fixative (1.6% paraformaldehyde, 2.5% glutaraldehyde, and 4% acetic acid in 0.1 M phosphate buffer [pH 7.4]). Fixative was replaced with saline before imaging with a Zeiss 510-Meta laser scanning confocal microscope. Images were rendered with Zeiss Zen software.

### Analysis of biofilm formation and architecture.

Z-stack images acquired at ×63 magnification with a Zeiss 510-Meta laser scanning confocal microscope were analyzed by COMSTAT2 to determine biomass (μm^3^/μm^2^), average thickness (μm), roughness (Ra), and percent area occupied by layers. Area occupied by layer was plotted as the percent bacterial biomass coverage per 1 μm optical section from the base of the biofilm. Standard error of the mean for replicate biofilms was calculated for each individual layer with Prism ver. 8.0 (GraphPad Software, San Diego, CA).

### Adherence of NTHi strain R2866 to Chang cells.

Chang cells (1 × 10^4^ cells) in 100 μl total volume were seeded into Transwell inserts with a 6.5-mm diameter and 0.4-um pore size (Corning Incorporated, Corning, New York). Cell culture medium (DMEM, 10% heat-inactivated calf serum, and 2 mM l-glutamine) was replaced daily until cells reached confluence at 2 to 3 days. The apical surface of the cells was rinsed twice with sterile DPBS. Strain R2866 strains were added to the apical surface of the Chang cells at a multiplicity of infection (MOI) of 100 in 50 μl of DPBS and incubated for 30 min at 37°C. The cells were rinsed twice with DPBS, incubated with 3 units of Alexa Fluor 594 phalloidin (Thermo Fisher Scientific) for 30 min, and rinsed twice. The membrane of the Transwell was then excised and mounted with ProLong glass antifade mountant with NucBlue stain (Thermo Fisher Scientific). Images were captured on a LSM 700 laser scanning microscope and rendered with Zen software (Zeiss).

### Adherence assays with BL21 strains.

E. coli BL21 strains expressing either wild-type Hia, D618K/A620R double mutant, R674A mutant, or containing the empty pET15b expression vector were grown to the mid-log phase (OD_600_, ∼0.6) in LB broth containing ampicillin (100 μg/ml), and CFU were calculated by serially diluting. Approximately 5 × 10^5^ CFU (100 μl) of each mid-log culture was added to wells of a 24-well plate containing differentiated Chang cells as described previously (23). Six wells were used per strain per experiment. Following the addition of BL21, plates were incubated at 37°C for 2 h. Following incubation, supernatant was removed and nonadherent cells were removed by gentle washing with 1× PBS four times. Adherent cells were released by incubating with 0.05% trypsin in 1× PBS for 10 min at room temperature. Adherent bacteria were quantified by serial dilution and plating. Percent adherence was calculated as the number of adherent CFU in relation to the total input CFU per strain. A one-way analysis of variance (ANOVA) was carried out using the total number of each output and comparing to wild-type cells. All data (total CFU and percent adherence) are presented as Data Set S1 in the supplemental material.

### Modeling interaction of Hia BD1 with 2-6 SLN.

Docking of 2-6 SLN to Hia BD1 was performed using the AutoDock Vina protocol (61), which has the highest scoring power among commercial and academic molecular docking programs (62) and is implemented in the YASARA Structure molecular modeling package (ver. 16.46) (63). The docking experiment was set up by using the X-ray crystal structure of Hia BD1 (PDB code 1S7M) (22) with a box centered on D620 and using a grid size of 50 Å × 50 Å × 50 Å (*x*, *y*, *z*) covering chain C. In total, 25 Vina docking runs were performed. The three-dimensional topology of the 2-6 SLN glycan was generated using the carbohydrate builder available at the GLYCAM-Web server (http://glycam.org).
