# Supplementary figures and images for: The Nontypeable Haemophilus influenzae Major Adhesin Hia Is a Dual-Function Lectin That Binds to Human-Specific Respiratory Tract Sialic Acid Glycan Receptors
Source: mBio. 2020 Nov 3;11(6):e02714-20. doi: 10.1128/mBio.02714-20 (PMC7642680; doi:10.1128/mBio.02714-20)

A)

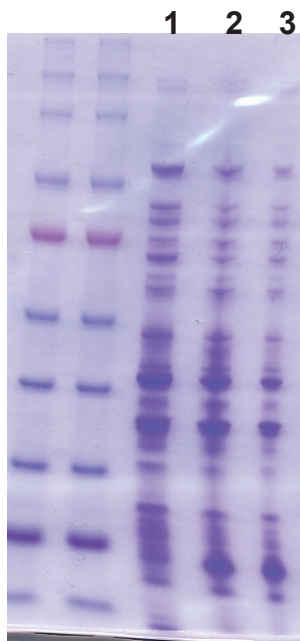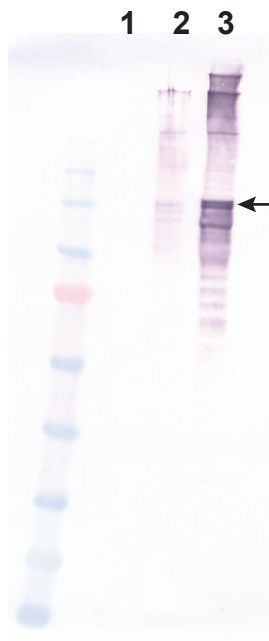

1. BL21 only
2. BL21 pET15b::Hia uninduced
3. BL21 pET15b::Hia induced

B)

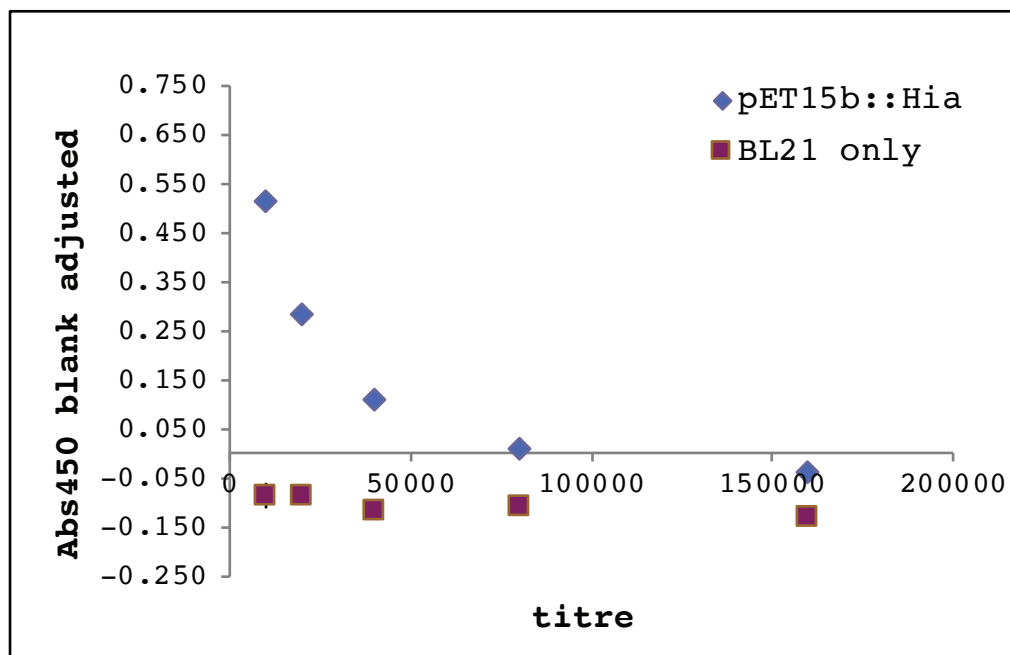

Supplement: FIG S1 [file mBio.02714-20-sf001.pdf]

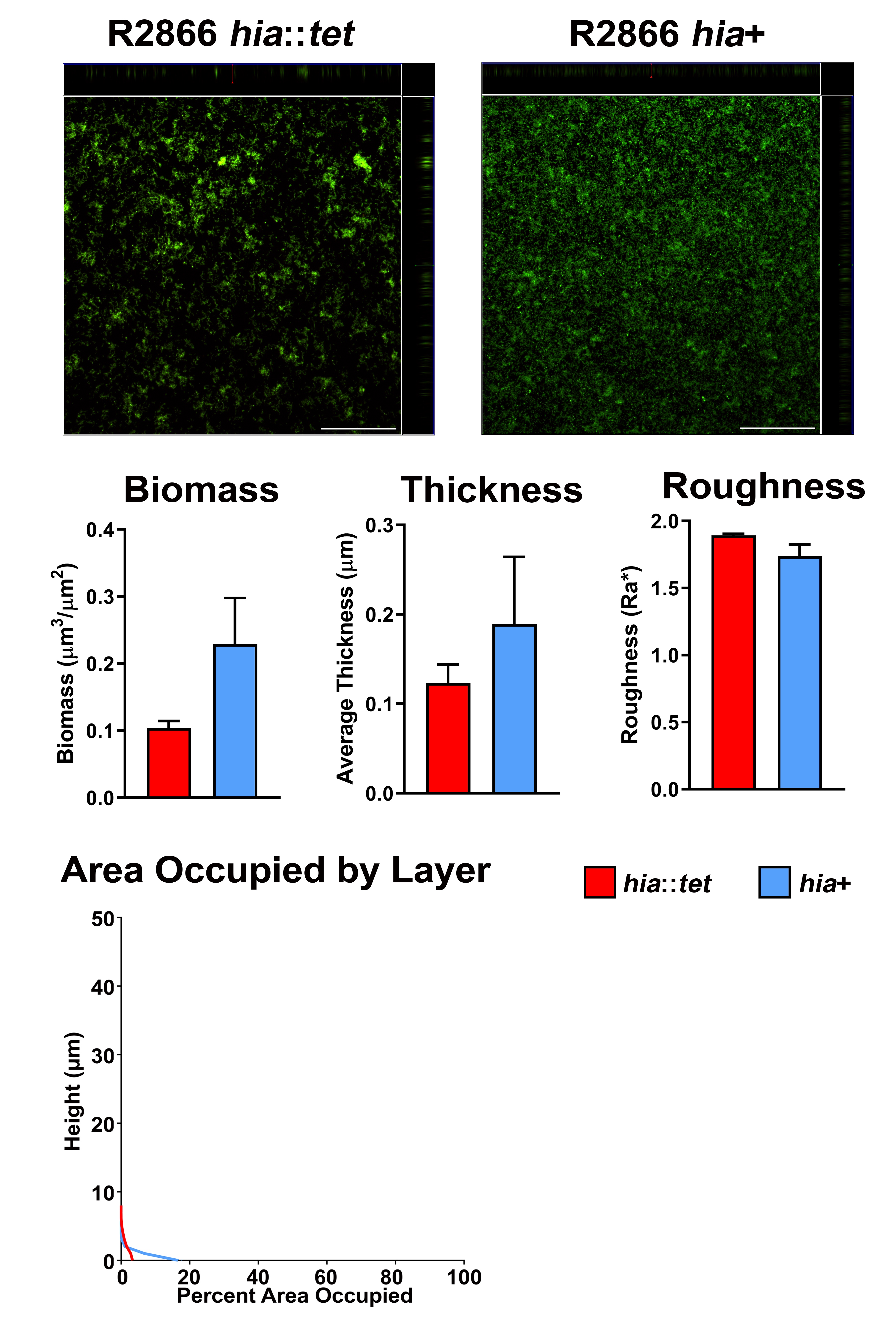

Supplement: FIG S2 [file mBio.02714-20-sf002.jpg]
