# Supplementary material for: The Nontypeable Haemophilus influenzae Major Adhesin Hia Is a Dual-Function Lectin That Binds to Human-Specific Respiratory Tract Sialic Acid Glycan Receptors
Source: mBio. 2020 Nov 3;11(6):e02714-20. doi: 10.1128/mBio.02714-20 (PMC7642680; doi:10.1128/mBio.02714-20)
Supplement: TABLE S1 [file mBio.02714-20-st001.docx]

**Supplementary Table 1 - Primers used in this study**

| **Primer** | **Sequence** |
| --- | --- |
| Hia-UP-F | ﻿GTAGAAAACTTAGCAACATTAAACGG |
| Hia-UP-R | CCA TTT TGA CCA TTA GCA TCG G |
| Hia-INV-F | ﻿GTTATTTGGAATGTTGTGACTCAAA |
| Hia-INV-R | GAA AAA CAA ACA TTT ACA CAA AAA TCA AAT ATT TTC |
| HiaFULL-F | AGTCAG TCATGA ACAAAATTTTTAACGTTATTTGGAATG |
| HiaFULL-R | AGTCAG CTCGAG TTACCACTGGTAACCAACACC |
| Hia-D618K-F | GACAA C TTA ACG AAA CAA AAT **AAA** GAT GCC TAT AAA GGC TTG ACC AAT TTG G |
| Hia-A620R-F | GACAA C TTA ACG AAA CAA AAT GAC GAT **CGC** TAT AAA GGC TTG ACC AAT TTG G |
| Hia-618/620-double-F | GACAA C TTA ACG AAA CAA AAT **AAA** GAT **CGC** TAT AAA GGC TTG ACC AAT TTG G |
| Hia-618/620-R | GAC GGA GCT AGT CAG CGG ATC GAA ATT CG |
| Hia-R674A-F | **GAA TAT CAC GAT CAA GTT GCC AAT GCG AAC GAA GTG AAA TTC** |
| Hia-R674A-R | **CGT TGA GCC GCC TGT GGT TTT GTC C** |
| Hia-screen-F | GGC AAG AAC TTA AAA GTG AAA CAA GAG G |
| Hia-screen-R | **CCA GAA CCT TTG TTG GTT ATG GTA GC** |
| HiaBD1-F | AGT CAG CATATG AAC AAC AAT ACT CCT GTT ACG AAT AAG TTG |
| HiaBD1-R | AGCTAG GGATCC GCC ATT TTG ACC ATT AGC ATC GGT TG |
