# Supplementary material for: The Nontypeable Haemophilus influenzae Major Adhesin Hia Is a Dual-Function Lectin That Binds to Human-Specific Respiratory Tract Sialic Acid Glycan Receptors
Source: mBio. 2020 Nov 3;11(6):e02714-20. doi: 10.1128/mBio.02714-20 (PMC7642680; doi:10.1128/mBio.02714-20)
Supplement: TABLE S2 [file mBio.02714-20-st002.pdf]

ID	Structure	Hia fold over BL21 control
	**MONOSACCHARIDES**	
1	Fucα-sp3	0.42
2	Galα-sp3	0.43
3	Galβ-sp3	0.60
4	GalNAcα-sp0	0.45
5	GalNAcα-sp3	0.37
6	GalNAcβ-sp3	0.40
7	Glcα-sp3	0.74
9	Glcβ-sp3	0.26
10	GlcNAcβ-sp3	0.30
14	GlcN(Gc)β-sp4	0.19
15	$HOCH_2(HOCH)_4CH_2NH_2$	0.47
16	Manα-sp3	0.51
18	Manβ-sp4	0.51
19	ManNAcβ-sp4	0.53
20	Rhaα-sp3	0.35
22	GlcNAcβ-sp4	0.25
37	3-O-Su-Galβ-sp3	0.41
38	3-O-Su-GalNAcα-sp3	0.50
43	6-O-Su-GlcNAcβ-sp3	0.44
44	GlcAα-sp3	0.33
45	GlcAβ-sp3	0.36
46	6-H_2PO_3Glcβ-sp4	0.43
47	6-H_2PO_3Manα-sp3	3.92±0.61
48	Neu5Acα-sp3	6.22±0.93
49	Neu5Acα-sp9	5.61±0.97
52	Neu5Gcα-sp3	0.34
54	9-NAc-Neu5Acα-sp3	0.54
55	3-O-Su-GlcNAcβ-sp3	0.60
	**Terminal Galactose**	
75	Galα1-2Galβ-sp3	1.05
76	Galα1-3Galβ-sp3	1.21
77	Galα1-3GalNAcβ-sp3	1.06
78	Galα1-3GalNAcα-sp3	0.97
80	Galα1-3GlcNAcβ-sp3	1.20
81	Galα1-4GlcNAcβ-sp3	1.08
83	Galα1-6Glcβ-sp4	1.06

84	Galβ1-2Galβ-sp3	0.99
85	Galβ1-3GlcNaAcβ-sp3	1.24
87	Galβ1-3Galβ-sp3	1.01
88	Galβ1-3GalNAcβ-sp3	1.13
89	Galβ1-3GalNAcα-sp3	1.23
93	Galβ1-4Glcβ-sp4	0.86
94	Galβ1-4Galβ-sp4	1.07
97	Galβ1-4GlcNAcβ-sp3	1.05
100	Galβ1-6Galβ-sp4	1.05
145	Galβ1-3(6-O-Su)GlcNAcβ-sp3	2.19±0.44
146	Galβ1-4(6-O-Su)Glcβ-sp2	1.24
147	Galβ1-4(6-O-Su)GlcNAcβ-sp3	2.41±0.39
150	3-O-Su-Galβ1-3GalNAcα-sp3	0.96
151	6-O-Su-Galβ1-3GalNAcα-sp3	0.90
152	3-O-Su-Galβ1-4Glcβ-sp2	1.02
153	6-O-Su-Galβ1-4Glcβ-sp2	0.94
155	3-O-Su-Galβ1-3GlcNAcβ-sp3	0.94
157	3-O-Su-Galβ1-4GlcNAcβ-sp3	0.88
159	4-O-Su-Galβ1-4GlcNAcβ-sp3	0.84
161	6-O-Su-Galβ1-3GlcNAcβ-sp3	0.98
163	6-O-Su-Galβ1-4GlcNAcβ-sp3	0.83
176	3-O-Su-Galβ1-4(6-O-Su)Glcβ-sp2	0.77
177	3-O-Su-Galβ1-4(6-O-Su)GlcNAcβ-sp2	0.97
178	6-O-Su-Galβ1-4(6-O-Su)Glcβ-sp2	1.00
179	6-O-Su-Galβ1-3(6-O-Su)GlcNAcβ-sp2	0.89
180	6-O-Su-Galβ1-4(6-O-Su)GlcNAcβ-sp2	0.63
181	3,4-O-Su_2-Galβ1-4GlcNAcβ-sp3	0.92
182	3,6-O-Su_2-Galβ1-4GlcNAcβ-sp2	0.92
183	4,6-O-Su_2-Galβ1-4GlcNAcβ-sp2	0.88
184	4,6-O-Su_2-Galβ1-4GlcNAcβ-sp3	1.18
189	3,6-O-Su_2-Galβ1-4(6-O-Su)GlcNAcβ-sp2	0.60
201	3,4-O-Su_2-Galβ1-4GlcNAcβ-sp3	0.91
203	Galβ1-4(6-O-Su)GlcNAcβ-sp2	0.87
220	Galα1-3Galβ1-4Glcβ-sp2	0.78
222	Galα1-3Galβ1-4GlcNAcβ-sp3	0.89
224	Galα1-4Galβ1-4Glcβ-sp3	0.89
225	Galα1-4Galβ1-4GlcNAc-sp2	0.93
228	Galβ1-2Galα1-4GlcNAcβ-sp4	0.88

229	Galβ1-3Galβ1-4GlcNAcβ-sp4	0.73
231	Galβ1-4GlcNAcβ1-3GalNAcα-sp3	0.77
232	Galβ1-4GlcNAcβ1-6GalNAcα-sp3	1.21
254	Galβ1-3(GlcNAcβ1-6)GalNAcα-sp3	1.16
262	Galβ1-3GalNAcβ1-3Gal-sp4	0.80
264	Galβ1-4Galβ1-4GlcNAc-sp3	0.99
373	Galα1-3Galβ1-4GlcNAcβ1-3Galβ-sp3	1.21
375	Galα1-4GlcNAcβ1-3Galβ1-4GlcNAcβ-sp3	0.96
376	Galβ1-3GlcNAcβ1-3Galβ1-4Glcβ-sp4	1.19
377	Galβ1-3GlcNAcβ1-3Galβ1-3GlcNAcβ-sp2	1.02
378	Galβ1-3GlcNAcα1-3Galβ1-4GlcNAcβ-sp3	1.20
379	Galβ1-3GlcNAcβ1-3Galβ1-4GlcNAcβ-sp3	1.24
380	Galβ1-3GlcNAcα1-6Galβ1-4GlcNAcβ-sp2	0.77
381	Galβ1-3GlcNAcβ1-6Galβ1-4GlcNAcβ-sp2	1.04
382	Galβ1-3GalNAcβ1-4Galβ1-4Glcβ-sp3	1.10
383	Galβ1-4GlcNAcβ1-3Galβ1-4Glcβ-sp2	1.14
385	Galβ1-4GlcNAcβ1-3Galβ1-4GlcNAcβ-sp3	1.05
387	Galβ1-4GlcNAcβ1-6Galβ1-4GlcNAcβ-sp2	1.12
388	Galβ1-4GlcNAcβ1-6(Galβ1-3)GalNAcα-sp3	1.14
504	(A-GN-M)$_2$-3,6-M-GN-GNβ-sp4	0.88
1A	Galβ1-3Glc*N* Ac	1.26
1B	Galβ1-4Glc*N* Ac	1.04
1C	Galβ1-4Gal	1.11
1D	Galβ1-6Glc*N* Ac	1.16
1E	Galβ1-3Gal*N* Ac	1.04
1F	Galβ1-3GalNAcβ1-4Galβ1-4Glc	1.11
1G	Galβ1-3Glc*N* Acβ1-3Galβ1-4Glc	0.91
1H	Galβ1-4Glc*N* Acβ1-3Galβ1-4Glc	1.10
1I	Galβ1-4Glc*N* Acβ1-6(Galβ1-4Glc*N* Acβ1-3)Galβ1-4Glc	1.05
1J	Galβ1-4Glc*N* Acβ1-6(Galβ1-3Glc*N* Acβ1-3)Galβ1-4Glc	1.02
1K	Gala1-4Galβ1-4Glc	0.99
1L	Gal*N* Acα1-*O* -Ser	1.04
1M	Galb1-3Gal*N* Acα1-*O* -Ser	1.02
1N	Gala1-3Gal	0.77
1O	Gala1-3Galβ1-4Glc*N* Ac	1.04
1P	Gala1-3Galβ1-4Glc	0.98
2A	Gala1-3Galβ1-4Galα1-3Gal	1.22
2B	Galβ1-6Gal	1.20

2C	Gal*N* Acβ1-3Gal	1.03
2D	Gal*N* Acβ1-4Gal	0.93
2E	Galα1-4Galβ1-4Glc*N* Ac	1.03
2F	GalNAcα1-3Galβ1-4Glc	1.01
2G	Galβ1-3Glc*N* Acβ1-3Galβ1-4Glc*N* Acβ1-6(Galβ1-3Glc*N* Acβ1-3)Galβ1-4Glc	0.82
2H	Galβ1-3Glc*N* Acβ1-3Galβ1-4Glc*N* Acβ1-3Galβ1-4Glc	0.99
18B	Galβ1-3GalNAcβ1-3Galα1-4Galβ1-4Glc	0.85
18C	Galβ1-3GalNAcβ1-3Gal	1.12
18L	Galβ1-4Glc	1.23
18M	Galβ1-4Gal	0.83
18N	Galβ1-6Gal	1.00
**Terminal N-Acetylgalactosamine**		
101	GalNAcα1-3GalNAcβ-sp3	1.05
102	GalNAcα1-3Galβ-sp3	0.95
103	GalNAcα1-3GalNAcα-sp3	1.17
104	GalNAcβ1-3Galβ-sp3	1.16
106	GalNAcβ1-4GlcNAcβ-sp3	1.12
192	GalNAcβ1-4(6-O-Su)GlcNAcβ-sp3	1.10
193	3-O-Su-GalNAcβ1-4GlcNAcβ-sp3	1.23
194	6-O-Su-GalNAcβ1-4GlcNAcβ-sp3	1.17
195	6-O-Su-GalNAcβ1-4-(3-O-Su)GlcNAcβ-sp3	0.81
196	3-O-Su-GalNAcβ1-4(3-O-Su)-GlcNAcβ-sp3	1.14
197	3,6-O-Su_2-GalNAcβ1-4GlcNAcβ-sp3	1.22
198	4,6-O-Su_2-GalNAcβ1-4GlcNAcβ-sp3	1.20
199	4,6-O-Su_2-GalNAcβ1-4-(3-O-Ac)GlcNAcβ-sp3	0.95
200	4-O-Su-GalNAcβ1-4GlcNAcβ-sp3	1.00
202	6-O-Su-GalNAcβ1-4(6-O-Su)GlcNAcβ-sp3	0.92
204	4-O-Su-GalNAcβ1-4GlcNAcβ-sp2	1.00
238	GalNAcβ1-4Galβ1-4Glcβ-sp3	1.03
389	GalNAcβ1-3Galα1-4Galβ1-4Glcβ-sp3	1.07
1L	Gal*N* Acα1-*O* -Ser	1.16
2C	Gal*N* Acβ1-3Gal	0.99
2D	Gal*N* Acβ1-4Gal	1.10
2F	GalNAcα1-3Galβ1-4Glc	1.21
**Fucosylated**		
71	Fucα1-2Galβ-sp3	1.08
72	Fucα1-3GlcNAcβ-sp3	0.78
73	Fucα1-4GlcNAcβ-sp3	1.10

215	Fucα1-2Galβ1-3GlcNAcβ-sp3	1.02
216	Fucα1-2Galβ1-4GlcNAcβ-sp3	1.01
217	Fucα1-2Galβ1-3GalNAcα-sp3	1.12
219	Fucα1-2Galβ1-4Glcβ-sp4	1.07
226	Fucα1-2(Galα1-3)Galβ-sp3	0.99
233	Galβ1-3(Fucα1-4)GlcNAcβ-sp3	0.92
234	Fucα1-3(Galβ1-4)GlcNAcβ-sp3	0.91
235	Fucα1-2(GalNAcα1-3)Galβ-sp3	1.10
287	3-O-Su-Galβ1-3(Fucα1-4)GlcNAcβ-sp3	0.95
288	Fucα1-3(3-O-Su-Galβ1-4)GlcNAcβ-sp3	1.03
359	Fucα1-2(Galα1-3)Galβ1-3GlcNAcβ-sp3	1.09
360	Fucα1-2(Galα1-3)Galβ1-4GlcNAcβ-sp3	1.19
362	Fucα1-2(Galα1-3)Galβ1-3GalNAcα-sp3	0.68
363	Fucα1-2(Galα1-3)Galβ1-3GalNAcβ-sp3	1.17
364	Fucα1-3(Galα1-3Galβ1-4)GlcNAcβ-sp3	0.86
366	Fucα1-2(GalNAcα1-3)Galβ1-3GlcNAcβ-sp3	0.98
368	Fucα1-2(GalNAcα1-3)Galβ1-4GlcNAcβ-sp3	1.12
371	Fucα1-2Galβ1-3(Fucα1-4)GlcNAcβ-sp3	0.82
372	Fucα1-3(Fucα1-2Galβ1-4)GlcNAcβ-sp3	0.90
392	Fucα1-2(GalNAcα1-3)Galβ1-3GalNAcα-sp3	1.15
479	Fucα1-2Galβ1-3GlcNAcβ1-3Galβ1-4Glcβ-sp4	1.06
480	Fucα1-2Galβ1-3GlcNAcβ1-3Galβ1-4GlcNAcβ-sp2	1.24
483	Fucα1-3(Fucα1-2 (Galα1-3)Galβ1-4)GlcNAcβ-sp3	0.83
496	Fucα1-2Galβ1-3(Fucα1-4)GlcNAcβ1-3Galβ1-4Glcβ-sp4	0.90
497	Fucα1-3(Fucα1-2Galβ1-4)GlcNAcβ1-3Galβ1-4Glcβ-sp4	1.00
538	Le^{x}1-6'(Le^{c}1-3')Lac-sp4	0.98
539	LacNAc1-6'(Le^{d}1-3')Lac-sp4	0.90
541	Le^{x}1-6'(Le^{d}1-3')Lac-sp4	1.08
542	Le^{c}Le^{x}1-6'(Le^{c}1-3')Lac-sp4	0.68
543	Le^{x}1-6'(Le^{b}1-3')Lac-sp4	0.86
7A	Fucα1-2Galβ1-3Glc*N* Acβ1-3Galβ1-4Glc	1.13
7B	Galβ1-3(Fuca1-4)Glc*N* Acβ1-3Galβ1-4Glc	0.83
7C	Galβ1-4(Fuca1-3)Glc*N* Acβ1-3Galβ1-4Glc	0.87
7D	Fucα1-2Galβ1-3(Fucα1-4)Glc*N* Acβ1-3Galβ1-4Glc	1.23
7E	Galβ1-3(Fucα1-4)Glc*N* Acβ1-3Galβ1-4(Fucα1-3)Glc	1.14
7F	Fucα1-2Gal	0.73
7G	Fucα1-2Galβ1-4Glc	1.00
7H	Galβ1-4(Fucα1-3)Glc	0.94

7I	Galβ1-4(Fucα1-3)Glc*N* Ac	0.90
7J	Galβ1-3(Fucα1-4)Glc*N* Ac	0.99
7K	Gal*N* Acα1-3(Fucα1-2)Gal	0.85
7L	Fucα1-2Galβ1-4(Fucα1-3)Glc	0.81
7M	Galβ1-3(Fucα1-2)Gal	0.91
7N	Fucα1-2Galβ1-4(Fucα1-3)Glc*N* Ac	1.17
7O	Fucα1-2Galβ1-3Glc*N* Ac	1.09
7P	Fucα1-2Galβ1-3(Fucα1-4)Glc*N* Ac	1.01
8A	SO_3-3Galβ1-3(Fucα1-4)Glc*N* Ac	0.99
8B	SO_3-3Galβ1-4(Fucα1-3)Glc*N* Ac	0.97
8C	Galβ1-3Glc*N* Acβ1-3Galβ1-4(Fucα1-3)Glc*N* Acβ1-3Galβ1-4Glc	0.76
8D	Galβ1-4(Fucα1-3)Glc*N* Acβ1-6(Galβ1-3Glc*N* Acβ1-3)Galβ1-4Glc	1.01
8E	Galβ1-4(Fucα1-3)Glc*N* Acβ1-6(Fucα1-2Galβ1-3Glc*N* Acβ1-3)Galβ1-4Glc	0.97
8F	Galβ1-4(Fucα1-3)Glc*N* Acβ1-6(Fucα1-2Galβ1-3(Fucα1-4)Glc*N* Acβ1-3)Galβ1-4Glc	0.90
8G	Galβ1-4GlcNAcβ1-3Galβ1-4(Fucα1-3)Glc	1.01
8H	Fucα1-2Galβ1-4(Fucα1-3)GlcNAcβ1-3Galβ1-4Glc	0.76
8I	Fucα1-3Galβ1-4GlcNAcβ1-3Galβ1-4(Fucα1-3)Glc	1.11
8J	Fuca1-2Galb1-4(Fuca1-3)GlcNAcb1-3(Fuca1-2)Galb1-4Glc	0.76
8K	Galβ1-4(Fucα1-3)GlcNAcβ1-6(Galβ1-4GlcNAcβ1-3)Galβ1-4Glc	1.17
8L	Galb1-4(Fuca1-3)GlcNAcb1-6(Galb1-4(Fuca1-3)GlcNAcb1-3)Galb1-4Glc	0.86
8M	Fuca1-2Galb1-4(Fuca1-3)GlcNAcb1-6(Galb1-4GlcNAcb1-3)Galb1-4Glc	0.98
8N	Galb1-3GlcNAcb1-3Galb1-4(Fuca1-3)GlcNAcb1-6(Galb1-3GlcNAcb1-3)Galb1-4Glc	0.66
8O	Fucα1-2Galβ1-3GlcNAcβ1-3Galβb1-4(Fucα1-3)GlcNAcβ1-6(Galβ1-3GlcNAcβ1-3)Galβ1-4Glc	0.94
8P	GalNAcb1-3(Fuca1-2)Galb1-4Glc	1.00
9A	Galb1-3(Fuca1-2)Galb1-4(Fuca1-3)Glc	1.22
9B	Galβ1-4Glc*N* Acβ1-6(Fucα1-2Galβ1-3Glc*N* Acβ1-3)Galβ1-4Glc	0.81
18D	Galα1-3(Fucα1-2)Galβ1-4Glc	0.96
18E	GalNAcα1-3(Fucα1-2)Galβ1-4(Fucα1-3)Glc	0.87
19J	Galβ1-4(Fucα1-3)GlcNAcβ1-3Gal	1.02
19L	Fucα1-2Galβ1-4(Fucα1-3)GlcNAcβ1-3Gal	0.93
19M	Galβ1-3(Fucα1-4)GlcNAcβ1-3Gal	0.87
19N	Fucα1-2Galβ1-3(Fucα1-4)GlcNAcβ1-3Gal	0.70
	**Sialylated**	
169	Neu5Acα2-3Galβ-sp3	1.29
170	Neu5Acα2-6Galβ-sp3	8.84±1.47
171	Neu5Acα2-3GalNAcα-sp3	1.30
172	Neu5Acα2-6GalNAcα-sp3	1.95
174	Neu5Gcα2-6GalNAcα-sp3	1.58

186	Neu5Acα2-8Neu5Acα2-sp3	1.78
205	Neu5Acα2-6GalNAcβ-sp3	9.34±1.69
206	Neu5Gcα2-3Gal-sp3	1.27
289	Galα1-3(Neu5Acα2-6)GalNAcα-sp3	1.36
290	Galβ1-3(Neu5Acα2-6) GalNAcα-sp3	1.19
292	Neu5Acα2-3Galβ1-3GalNAcα-sp3	1.60
293	Neu5Acα2-3Galβ1-4Glcβ-sp3	1.59
294	Neu5Acα2-3Galβ1-4Glcβ-sp4	1.69
295	Neu5Acα2-6Galβ1-4Glcβ-sp2	1.56
298	Neu5Acα2-3Galβ1-4GlcNAcβ-sp3	3.34±0.89
299	Neu5Acα2-3Galβ1-3GlcNAcβ-sp3	1.81
300	Neu5Acα2-6Galβ1-4GlcNAcβ-sp3	1.82
303	Neu5Gcα2-3Galβ1-4GlcNAcβ-sp3	1.33
304	Neu5Gcα2-6Galβ1-4GlcNAcβ-sp3	1.45
306	9-NAc-Neu5Acα2-6Galβ1-4GlcNAcβ-sp3	1.29
315	Neu5Acα2-3Galβ1-4-(6-O-Su)GlcNAcβ-sp3	1.84
317	Neu5Acα2-3Galβ1-3-(6-O-Su)GalNAcβ-sp3	1.57
318	Neu5Acα2-6Galβ1-4-(6-O-Su)GlcNAcβ-sp3	9.44±2.21
319	Neu5Acα2-3-(6-O-Su)Galβ1-4GlcNAcβ-sp3	4.37±0.67
321	(Neu5Acα2-8)$_3$-sp3	1.38
323	Neu5Acα2-6Galβ1-3GlcNAc-sp3	1.20
324	Neu5Acα2-6Galβ1-3(6-O-Su)GlcNAc-sp3	1.32
331	Neu5Gcα2-3Galβ1-3GlcNAcβ-sp3	1.48
421	Neu5Acα2-3(GalNAcβ1-4)Galβ1-4Glcβ-sp2	1.26
422	Neu5Acα2-3Galβ1-4GlcNAcβ1-3Galβ-sp3	1.33
423	Fucα1-3(Neu5Acα2-3Galβ1-4)GlcNAcβ-sp3	
426	Neu5Acα2-3Galβ1-3(Fucα1-4)GlcNAcβ-sp3	1.28
428	Fucα1-3(Neu5Acα2-3Galβ1-4)6-O-Su-GlcNAcβ-sp3	1.40
429	Fucα1-3(Neu5Acα2-3(6-O-Su)Galβ1-4)GlcNAcβ-sp3	1.39
433	Neu5Acα2-3Galβ1-3(Neu5Acα2-6)GalNAcα-sp3	1.31
434	Neu5Acα2-8Neu5Acα2-3Galβ1-4Glcβ-sp4	1.22
527	Neu5Acα2-3Galβ1-4GlcNAcβ1-3Galβ1-4GlcNAcβ-sp2	1.34
528	Fucα1-3(Neu5Acα2-3 Galβ1-4)GlcNAcβ1-3Galβ-sp3	4.33±1.12
529	Neu5Acα2-6(Galβ1-3)GlcNAcβ1-3Galβ1-4Glcβ-sp4	1.46
531	GalNAcβ1-4(Neu5Acα2-8Neu5Acα2-3)Galβ1-4Glc-sp2	1.29
532	Neu5Acα2-8Neu5Acα2-8Neu5Acα2-3Galβ1-4Glc-sp2	1.48
533	(Neu5Acα2-8)2Neu5Acα2-3(GalNAcβ1-4)Galβ1-4Glc-sp2	1.55
534	Neu5Acα2-3Galβ1-4GlcNAcβ1-3Galβ1-4GlcNAcβ-sp3	1.47

536	Neu5Acα2-3Galβ1-3GlcNAcβ1-3Galβ1-4Glcβ-sp4	1.86
537	Neu5Acα2-3Galβ1-4GlcNAcβ1-3Galβ1-4Glcβ-sp4	1.77
540	Le^x1-6'(6'SLN1-3')Lac-sp4	1.56
10A	Neu5Acα2-3Galβ1-3(Fucα1-4)Glc*N* Ac	1.62
10B	Neu5Acα2-3Galβ1-4(Fucα1-3)Glc*N* Ac	1.62
10C	Neu5Acα2-3Galβ1-3Glc*N* Acβ1-3Galβ1-4Glc	1.66
10D	Galβ1-4(Fucα1-3)Glc*N* Acβ1-6(Neu5Acα2-6Galβ1-4Glc*N* Acβ1-3)Galβ1-4Glc	1.45
10E	Neu5Acα2-3Galβ1-3(Neu5Acα2-6)GalNAc	1.33
10H	Neu5Acα2-6Galβ1-3GlcNAcβ1-3Galβ1-4(Fucα1-3)Glc	7.47±1.42
10I	Galβ1-3GlcNAcβ1-3(Neu5Acα2-6Galβ1-4GlcNAcβ1-6)Galβ1-4Glc	1.51
10J	Neu5Acα2-6Galβ1-3GlcNAcβ1-3(Galβ1-4GlcNAcβ1-6)Galβ1-4Glc	1.59
10K	Neu5Acα2-3Galβ1-4Glc*N* Ac	1.18
10L	Neu5Acα2-6Galβ1-4Glc*N* Ac	11.56±2.37
10M	Neu5Acα2-3Galβ1-3GlcNAcβ1-3Galβ1-4Glc	1.49
10N	Galβ1-3(Neu5Acα2-6)Glc*N* Acβ1-3Galβ1-4Glc	1.32
10O	Neu5Acα2-6Galβ1-4Glc*N* Acβ1-3Galβ1-4Glc	1.97
10P	Neu5Acα2-3Galβ1-3(Neu5Acα2-6)Glc*N* Acβ1-3Galβ1-4Glc	1.80
11A	Neu5Acα2-3Galβ1-4Glc	1.67
11B	Neu5Acα2-6Galβ1-4Glc	1.94
11C	(Neu5Acα2-8Neu5Ac)n (n<50)	1.29
11D	Neu5Acα2-6Galβ1-4GlcNAcβ1-2Manα1-6(Neu5Acα2-6Galβ1-4GlcNAcβ1-2Manα1-6)Manβ1-4GlcNAcβ1-4GlcNAc-Asn	1.90
18A	Neu5Acα2-3Galβ1-4GlcNAcβ1-3Galβ1-4Glc	1.93
18K	9-NAc-Neu5Ac	1.63
18O	Neu5Gc	1.32
19K	Neu5Acα2-3Galβ1-4(Fucα1-3)GlcNAcβ1-3Gal	1.24
**Mannose**		
119	Manα1-2Manβ-sp4	0.79
120	Manα1-3Manβ-sp4	1.17
121	Manα1-4Manβ-sp4	0.94
122	Manα1-6Manβ-sp4	1.24
123	Manβ1-4GlcNAcβ-sp4	1.48
124	Manα1-2Manα-sp4	0.73
258	Manα1-3(Manα1-6)Manβ-sp4	1.38
495	Manα1-3(Manα1-3(Manα1-6)Manα1-6)Manβ-sp4	0.92
5A	Glc*N* Acβ1-2Man	1.36
5B	Glc*N* Acβ1-2Manα1-6(Glc*N* Acβ1-2Manα1-3)Man	1.39
5C	Manα1-2Man	0.92
5D	Manα1-3Man	1.03

5E	Manα1-4Man	0.95
5F	Manα1-6Man	1.48
5G	Manα1-6(Manα1-3)Man	0.98
5H	Manα1-6(Manα1-3)Manα1-6(Manα1-3)Man	0.93
**Terminal N-Acetylglucosamine**		
113	GlcNAcβ1-3GalNAcα-sp3	0.84
114	GlcNAcβ1-3Manβ-sp4	1.04
115	GlcNAcβ1-4GlcNAcβ-Asn	0.84
117	GlcNAcβ1-4GlcNAcβ-sp4	0.94
118	GlcNAcβ1-6GalNAcα-sp3	0.83
149	GlcNAcβ1-4(6-O-Su)GlcNAcβ-sp2	0.85
167	GlcNAcβ1-4-[HOOC(CH_3)CH]-3-O-GlcNAcβ-sp4	1.05
168	GlcNAcβ1--[HOOC(CH_3)CH]-3-O-GlcNAcβ-L-alanyl-D-i-glutaminyl-L-lysine	0.92
246	GlcNAcβ1-2Galβ1-3GalNAcα-sp3	0.87
247	GlcNAcβ1-3Galβ1-3GalNAcα-sp3	0.82
248	GlcNAcβ1-3Galβ1-4Glcβ-sp2	0.93
250	GlcNAcβ1-3Galβ1-4GlcNAcβ-sp3	0.96
251	GlcNAcβ1-4Galβ1-4GlcNAcβ-sp2	1.08
252	GlcNAcβ1-4GlcNAcβ1-4GlcNAcβ-sp4	0.81
253	GlcNAcβ1-6Galβ1-4GlcNAcβ-sp2	0.70
255	GlcNAcβ1-3(GlcNAcβ1-6)GalNAcα-sp3	1.03
395	GlcNAcβ1-3(GlcNAcβ1-6)Galβ1-4GlcNAcβ-sp3	1.14
493	(GlcNAcβ1-4)$_5$β-sp4	1.04
503	(GlcNAcβ1-4)$_6$β-sp4	1.09
505	(GN-M)$_2$-3,6-M-GN-GNβ-sp4	1.21
4A	Glc*N* Acβ1-4Glc*N* Ac	1.06
4B	Glc*N* Acβ1-4Glc*N* Acβ1-4Glc*N* Ac	0.93
4C	Glc*N* Acβ1-4Glc*N* Acβ1-4Glc*N* Acβ1-4Glc*N* Ac	0.74
4D	Glc*N* Acβ1-4Glc*N* Acβ1-4Glc*N* Acβ1-4Glc*N* Acβ1-4Glc*N* Acβ1-4Glc*N* Ac	0.95
4E	Bacterial cell wall muramyl discaccharide	0.89
4F	Glc*N* Acβ1-4Glc*N* Acβ1-4Glc*N* Acβ1-4Glc*N* Acβ1-4Glc*N* Ac	0.97
18G	6-O-Su-GlcNAc	0.73
18H	GlcNAc	1.06
**Glucose**		
110	Glcα1-4Glcβ-sp3	1.24
111	Glcβ1-4Glcβ-sp4	1.03
112	Glcβ1-6Glcβ-sp4	0.96
164	GlcAβ1-3GlcNAcβ-sp3	0.93

165	GlcAβ1-3Galβ-sp3	0.85
166	GlcAβ1-6Galβ-sp3	0.85
240	$(Glc\alpha1\text{-}4)_3\beta$-sp4	0.97
241	$(Glc\alpha1\text{-}6)_3\beta$-sp4	0.90
390	$(Glc\alpha1\text{-}4)_4\beta$-sp4	0.76
391	$(Glc\alpha1\text{-}6)_4\beta$-sp4	0.87
492	$(Glc\alpha1\text{-}6)_5\beta$-sp4	0.87
502	$(Glc\alpha1\text{-}6)_6\beta$-sp4	0.66
18I	GlcA	0.84
18J	6-O-(H_2PO_4)-Glc	1.08
19O	Glcα1-4Glcα1-4	0.84
19P	Glcα1-4Glcα1-4Glcα1-4	0.84
**Low molecular weight Carageenan and Glycoaminoglycans (GAGS)**		
12A	Neocarratetraose-41, 3-di-O-sulphate (Na+)	0.93
12B	Neocarratetraose-41-O-sulphate (Na+)	2.36±0.67
12C	Neocarrahexaose-24,41, 3, 5-tetra-O-sulphate (Na+)	1.08
12D	Neocarrahexaose-41, 3, 5-tri-O-sulphate (Na+)	2.43±0.71
12E	Neocarraoctaose-41, 3, 5, 7-tetra-O-sulphate (Na+)	0.73
12F	Neocarradecaose-41, 3, 5, 7, 9-penta-O-sulphate (Na+)	1.03
12G	ΔUA-2S-GlcNS-6S	1.05
12H	ΔUA-GlucNS-6S	0.96
12I	ΔUA-2S-GlucNS	0.86
12J	ΔUA-2S-GlcNAc-6S	0.93
12K	ΔUA-GlcNAc-6S	2.13±0.56
12L	ΔUA-2S-GlcNAc	0.95
12M	ΔUA-GlcNAc	1.16
12N	ΔUA-GalNAc-4S (Delta Di-4S)	1.27
12O	ΔUA-GalNAc-6S (Delta Di-6S)	0.78
12P	ΔUA-GalNAc-4S,6S (Delta Di-disE)	0.68
13A	ΔUA-2S-GalNAc-4S (Delta Di-disB)	0.90
13B	ΔUA-2S-GalNAc-6S (Delta Di-disD)	0.86
13C	ΔUA-2S-GalNAc-4S-6S (Delta Di-tisS)	0.73
13D	ΔUA-2S-GalNAc-6S (Delta Di-UA2S)	0.87
13E	ΔUA-GlcNAc (Delta Di-HA)	0.81
14M	ΔUA→2S-GlcN-6S	4.32±0.86
14N	ΔUA→GlcN-6S	0.89
14O	ΔUA→2S-GlcN	0.95
14P	ΔUA→GlcN	0.92

	**High molecular weight Carageenan and Glycoaminoglycans (GAGS)**	
625	(GlcAβ1-4GlcNAcβ1-3)$_{8}$-NH_2-ol	1.02
13F	(GlcAβ1-3Glc*N* Acβ1-4)n (n=4)	1.02
13G	(GlcAβ1-3Glc*N* Acβ1-4)n (n=8)	1.11
13H	(GlcAβ1-3Glc*N* Acβ1-4)n (n=10)	1.07
13I	(GlcAβ1-3Glc*N* Acβ1-4)n (n=12)	2.49±0.52
13J	(GlcA/IdoAα/β1-4Glc*N* Acα1-4)n (n=200)	0.88
13K	(GlcA/IdoAβ1-3(±4/6S)Gal*N* Acβ1-4)n (n<250)	3.78±0.88
13L	((±2S)GlcA/IdoAα/b1-3(±4S)Gal*N* Acβ1-4)n (n<250)	0.78
13M	(GlcA/IdoAβ1-3(±6S)Gal*N* Acβ1-4)n (n<250)	1.11
13N	HA - 4	2.21±0.91
130	HA - 6	1.40
13P	HA - 8	0.97
14A	HA 10	0.30
14B	HA-12	0.83
14C	HA-14	1.37
14D	HA-16	1.43
14E	HA 30000 da	1.35
14F	HA 107000 da	0.78
14G	HA 190000 da	0.60
14H	HA 220000 da	0.71
14I	HA 1600000 da	0.86
14J	Heparin sulfate	4.56±1.28
14K	β1-3Glucan	1.01
	**Complex N-glycans**	
627	(Sia2-6A-GN-M)$_2$-3,6-M-GN-GNβ-sp4	1.69
19A	Galβ1-4GlcNAcβ1-2Manα1-3(Galβ1-4GlcNAcβ1-2Manα1-6Man)β1-4GlcNAcβ1-4(Fucα1-6)GlcNAc	1.19
19B	Galβ1-4GlcNAcβ1-2(Galβ1-4GlcNAcβ1-4)Manα1-3(Galβ1-4GlcNAcβ1-2(Galβ1-4GlcNAcβ1-6)Manα1-6Man)β1-4GlcNAcβ1-4GlcNAc	0.97
19C	Neu5Acα2-6Galβ1-4GlcNAcβ1-2Manα1-3(Galβ1-4GlcNAcβ1-2Manα1-6)Manβ1-4GlcNAcβ1-4GlcNAc	1.52
19D	Neu5Acα2-6Galβ1-4GlcNAcβ1-2Manα1-3(Neu5Acα2-6Galβ1-4GlcNAcβ1-2Manα1-6)Manβ1-4GlcNAcβ1-4GlcNAc	1.58
19E	Galβ1-4GlcNAcβ1-2Manα1-3(Galβ1-4GlcNAcβ1-2Manα1-6)Manβ1-4GlcNAcβ1-4GlcNAc	0.84
19F	Neu5Acα2-6Galβ1-4GlcNAcβ1-2Manα1-3(Neu5Acα2-6Galβ1-4GlcNAcβ1-2Manα1-6)Manβ1-4GlcNAcβ1-4(Fucα1-6)GlcNAc	1.82
19G	Neu5Acα2-6Galβ1-4GlcNAcβ1-2(Neu5Acα2-6Galβ1-4GlcNAcβ1-4)Manα1-3(Neu5Acα2-6Galβ1-4GlcNAcβ1-2Manα1-6)Manβ1-4GlcNAcβ1-4Glc	1.93
19H	GlcNAcβ1-2(GlcNAcβ1-4)Manα1-3(GlcNAcβ1-2Manα1-6)GlcNAcβ1-4Manβ1-4GlcNAcβ1-4GlcNAc	0.61

Red/blue indicates binding. Binding is determined by positive interaction in three replicate array experiments. Positive interactions are determined by a background subtracted fluorescence value significantly above (greater than 2-fold) BL21 not expressing Hia protein.
